# Supplementary material for: Rational construction of a reversible arylazo-based NIR probe for cycling hypoxia imaging in vivo
Source: Nat Commun. 2021 May 13;12:2772. doi: 10.1038/s41467-021-22855-0 (PMC8119430; doi:10.1038/s41467-021-22855-0)
Supplement: Supplementary file 1 — Supplementary Information [file 41467_2021_22855_MOESM1_ESM.pdf]

Supplementary Information

For

Rational Construction of a Reversible Arylazo-based NIR Probe for Cycling Hypoxia Imaging *in vivo*

Yuming Zhang, Wenxuan Zhao, Yuncong Chen,* Hao Yuan, Hongbao Fang, Shankun Yao, Changli Zhang, Hongxia Xu, Nan Li, Zhipeng Liu, Zijian Guo,* Qingshun Zhao,* Yong Liang and Weijiang He*

1. Materials and general methods

All the reagents and solvents were purchased from commercial source including Aldrich, Alfa Aesar, JKchemical, and Energy Chemical. They were of analytic grade and used without purification except specified. Mito-Tracker Green, LysoSensor Green DND-189 and Image-iT™ Green hypoxia reagent were purchased from Invitrogen. Rat liver microsomes were purchased from Corning Gentest. NADPH was purchased from Sigma-Aldrich. DMPO was purchased from Dojindo. MEM media was purchased from Jiangsu Keygen BioTech Co. Ltd. AneroPack® and the matching culture bags and box were purchased from Mitsubishi Gas Chemical Company, Inc. Solutions of chemical species to test the sensing interference of HDSF were freshly prepared by dissolving $NaNO_3$, KNO_3, $Mg(NO_3)_2$, $Ca(NO_3)_2$, $Zn(NO_3)_2$, $Cu(NO_3)_2$, $Fe(NO_3)_3$, $Fe(SO_4)_2$, NH_4NO_3, $Ca(ClO)_2$, NaHS, L-cysteine, glutathione, homocysteine, H_2O_2, oxalic acid, ascorbic acid, sodium nitroferricyanide(III) with doubly distilled water. Superoxide anion radical ($O_2^{-\bullet}$), hydroxyl radical (•OH), and peroxynitrite (ONO_2^-) were generated from on-line reactions. $O_2^{-\bullet}$ was prepared by dissolving KO_2 in dry DMSO, and then sonication for 10 min. •OH was prepared through Fenton reaction (Fe^{2+}: H_2O_2 = 1: 10). ONO_2^- was generated by adding HCl to a mixture of $NaNO_2$ and H_2O_2 under 0 °C, before NaOH was quickly added in 1-2 sec to make a base solution. Content of ONO_2^- was determined by absorbance at 302 nm ($C = A_{302nm}/1.67$, mM), and solution was diluted with 0.1 M NaOH. All solutions used for hypoxia tests were conducted by bubbling pure argon gas until O_2 content was under 0.1 mg L^{-1}. *In vitro* O_2 content in solution was detected by Hach HQ30d with LDO101 O_2 probe. ^1H NMR and ^{13}C NMR spectra were recorded on Bruker DRX-400 with TMS as internal reference. High resolution mass spectrometric data were determined with an Agilent 6540Q-TOF LC/MS mass spectrometer, while ESI mass spectrometric data were determined with a Thermo Fisher ESI Mass Spectrometer. Fluorescence measurements were performed on FluoroMax-4 Spectrofluorometer with 5 nm slit for both excitation and emission. Absorption spectra were measured on a Perkin Elmer Lambda 35 spectrophotometer. All pH measurements were accomplished by a Model PHS-3C meter. Confocal imaging of MCF-7 cells and zebrafish embryos was realized using confocal microscope Zeiss LSM710. Optical imaging of mice was

carried out on PerkinElmer IVIS Lumina K Series III *in vivo* imaging system. Doppler ultrasound imaging was performed on VisualSonics Vevo2100.

2. Synthesis and characterization of HDMA and HDSF

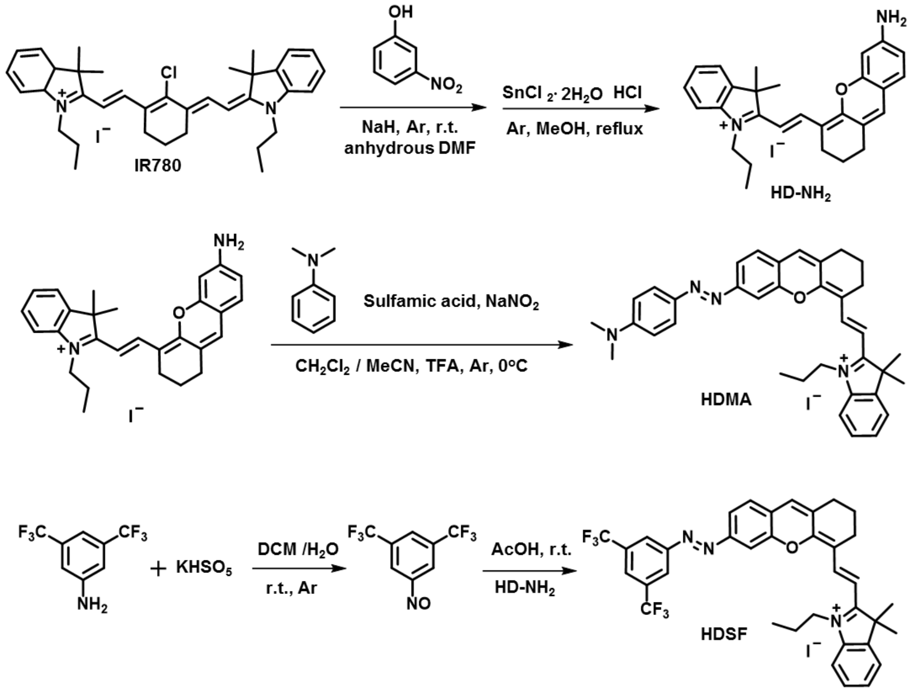

Supplementary Fig. 1 Synthesis of probes HDMA and HDSF.

Synthesis of HD-NH_2[1]

^{1}H NMR (400 MHz, CD_3OD): δ 8.63 (d, J = 14.3 Hz, 1H), 7.57-7.55 (m, 2H), 7.47-7.43 (m, 1H), 7.38-7.30 (m, 3H), 6.78-6.72 (m, 2H), 6.26 (d, J = 14.3 Hz, 1H), 4.18 (t, J = 7.3 Hz, 2H), 2.82-2.75 (m, 2H), 2.71 (t, J = 6.1 Hz, 2H), 1.95-1.87 (m, 4H), 1.78 (s, 6H), 1.06 (t, 7.4, 3H).

Synthesis of HDMA[2]

^{1}H NMR (400 MHz, CD_3OD): δ 8.53 (d, J = 15.1 Hz, 1H), 7.74-7.68 (m, 4H), 7.53-7.44 (m, 5H), 7.24(s, 1H), 6.69 (d, J = 9.1 Hz, 2H), 6.31 (d, J = 15.1 Hz, 1H), 4.15 (t, J = 7.4 Hz, 2H), 3.11(s, 6H), 2.71(t, 2H), 2.51 (t, J = 5.7 Hz, 2H), 1.91-1.83 (m, 10H), 1.04 (t, J = 7.4 Hz, 3H). ^{13}C NMR (101 MHz, CD_3OD): δ 179.68, 161.45, 156.14, 154.89, 154.71, 147.09, 144.53, 143.63, 142.74, 133.06, 131.91, 130.31, 129.46, 128.95, 126.83, 124.12, 123.89, 121.95, 119.59, 116.68, 116.27, 114.38, 112.70, 108.31, 106.34, 66.93, 52.33, 47.96, 40.44, 30.34, 28.33, 24.84, 22.37, 21.38, 15.48, 11.61. HR-MS (positive mode, m/z): Calcd. 543.3118, found 543.3111 for $[M]^+$.

Synthesis of probe HDSF

To a solution of 3,5-bis(trifluoromethyl)aniline (11.15 mmol, 2.54 g) in CH_2Cl_2 (75 mL), aqueous solution of oxone (22.3 mmol, 13.712 g) in water (112 mL) was added slowly. Then the mixture was stirred vigorously at room temperature for 22 h. The water layer was separated and extracted with CH_2Cl_2 (50 mL x2). The combined organic layer was washed with 0.1 M HCl (50 mL x2) and water (50 mL x2) successively, and dried over anhydrous Na_2SO_4. Solvent was removed under reduced pressure and resulted in green oil, which was used directly to reaction with compound 2 due to its instability. Compound 2 (0.7 mmol, 380 mg) dissolved in acetic acid (260 mL) was added into the green oil, and the mixture was stirred at room temperature in the

dark, and the reaction was monitored by TLC. After the reaction being fulfilled, solvent was removed under reduced pressure and the crude product was purified by silica gel chromatography with eluent MeCN/H_2O (50:1, v/v) to afford HDSF as a blue solid (56 mg). Yield, 10%.

^{1}H NMR (400 MHz, CD_3OD): δ 8.83 (d, J = 15.2 Hz, 1H), 8.51 (s, 2H), 8.19 (s, 1H), 8.02-7.90 (m, 2H), 7.74 (d, J = 7.1 Hz, 1H), 7.68 (d, J = 7.9 Hz, 2H), 7.61-7.50 (m, 2H), 7.33 (s, 1H), 6.73 (d, J = 15.3 Hz, 1H), 4.45 (t, J = 7.4 Hz, 2H), 2.84 (t, J = 6.1 Hz, 2H), 2.76 (t, J = 6.1 Hz, 2H), 1.99 (q, J = 7.4, 7.0 Hz, 4H), 1.89 (s, 6H), 1.10 (t, J = 7.4 Hz, 3H). ^{13}C NMR (101 MHz, CD_3OD): δ 181.04, 160.53, 154.49, 154.38, 147.75, 144.14, 142.73, 134.61, 134.29, 134.29, 133.93, 133.60, 131.43, 130.83, 130.44, 129.60, 129.51, 127.08, 125.88, 125.61, 124.28, 123.99, 123.17, 122.65, 116.90, 114.90, 110.33, 107.93, 52.77, 48.23, 30.57, 28.04, 25.00, 22.63, 21.46, 11.56. HR-MS (positive mode, m/z): Calcd. 636.2444, found 636.2478 for $[M]^+$.

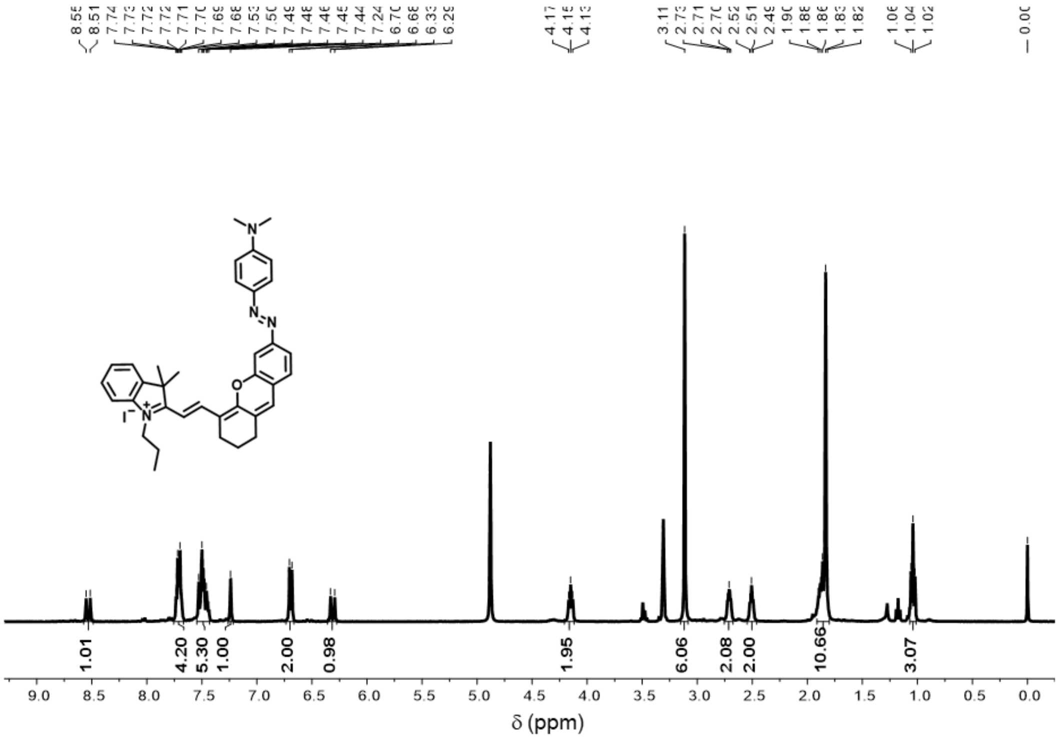

Supplementary Fig. 2 ^{1}H NMR spectrum of HDMA in CD_3OD.

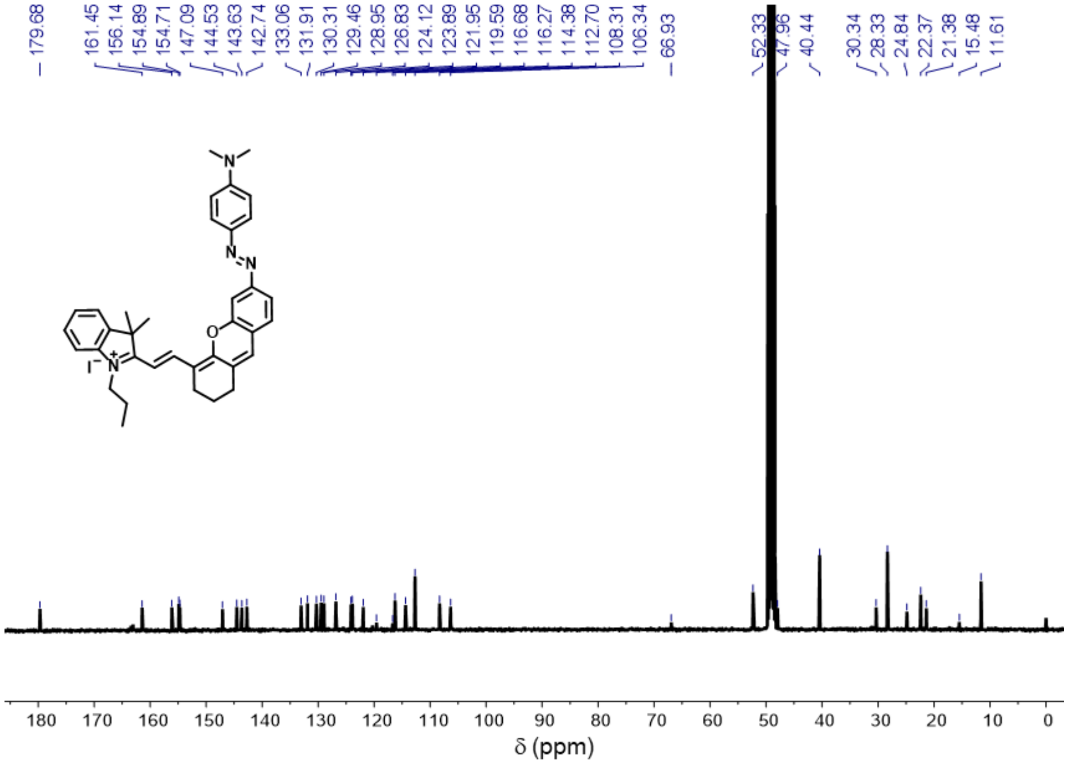

Supplementary Fig. 3 ^{13}C NMR spectrum of HDMA in CD_3OD.

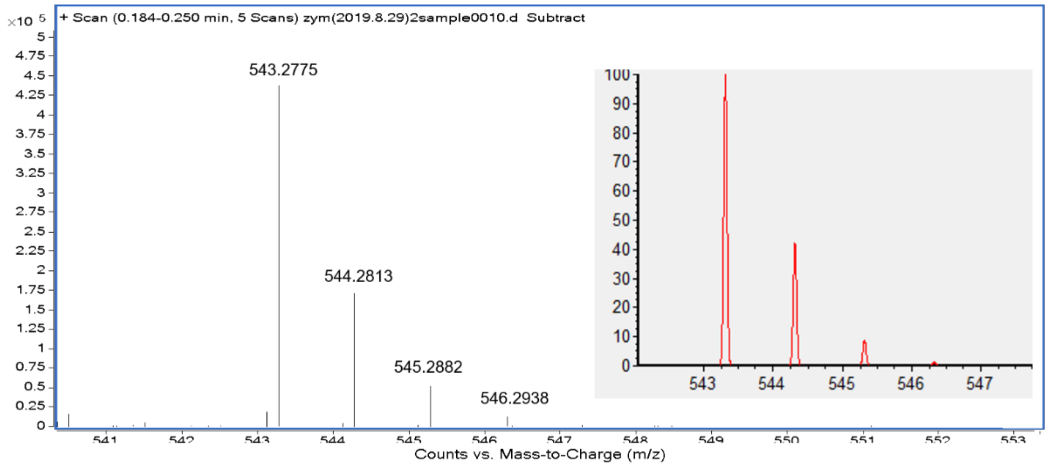

Supplementary Fig. 4 HR-MS spectrum of HDMA. Inset: Simulated isotopic distribution pattern of HDMA.

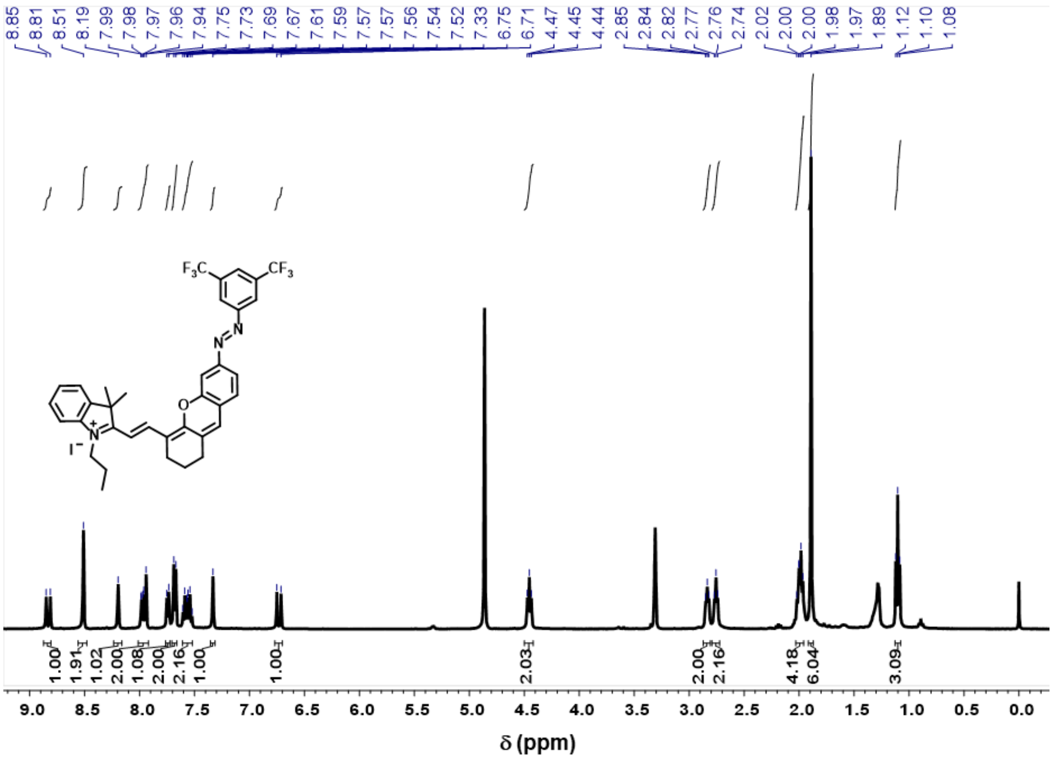

Supplementary Fig. 5 ^1H NMR spectrum of HDSF in CD_3OD.

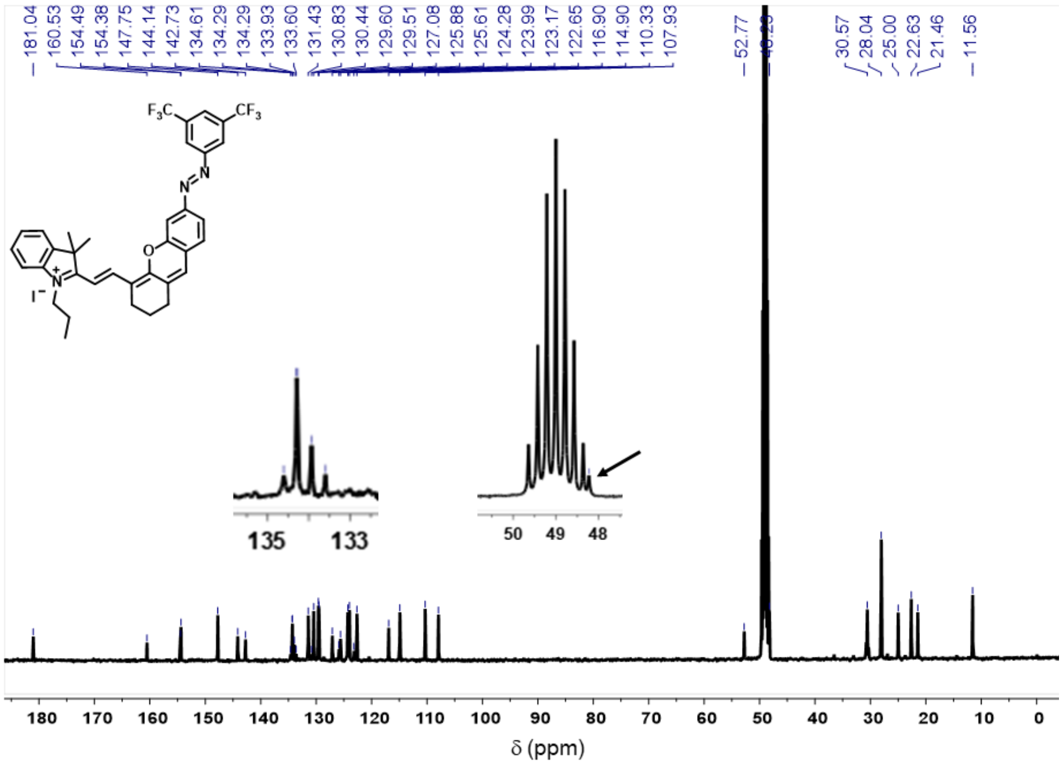

Supplementary Fig. 6 ^{13}C NMR spectrum of HDSF in CD_3OD.

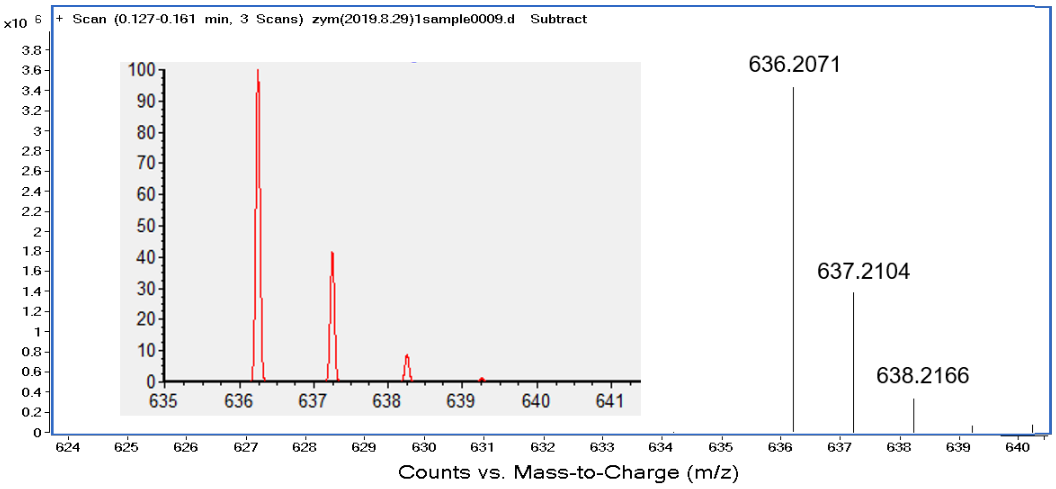

Supplementary Fig. 7 HR-MS spectrum of HDSF. Inset: Simulated isotopic distribution pattern of HDMA.

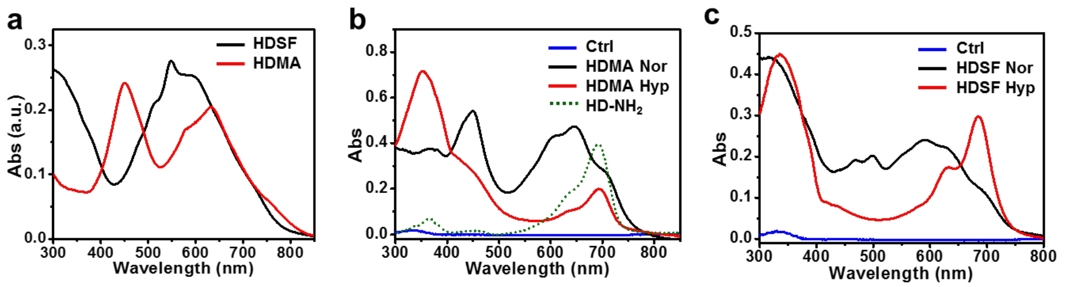

Supplementary Fig. 8 Absorption spectra of 20 μM HDMA and HDSF in PBS buffer (0.1 M, pH 7.4, 2% DMSO, v/v), (a) without RLM or NADPH, (b, c) with the existence of rat liver microsomes (RLM, 250 μg mL^{-1}) and NADPH (100 μM) incubated at 37 °C for 30 min in normoxic (Nor) and hypoxic (Hyp) conditions respectively. Ctrl: PBS buffer containing RLM and NADPH. The control solution was used as the reference in UV-Vis absorption tests in (b) and (c).

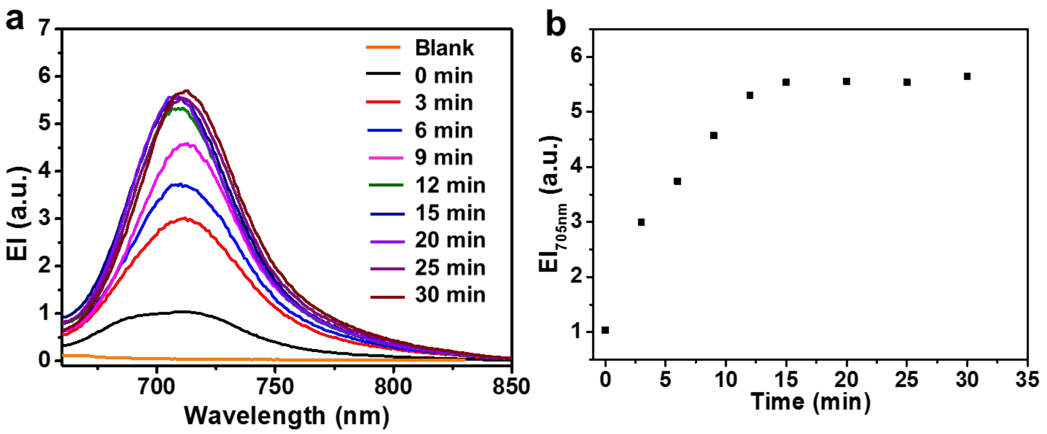

Supplementary Fig. 9 (a) Time-dependent emission spectra of 20 μM HDSF in PBS buffer (0.1 M, pH 7.4, 2% DMSO, v/v) containing rat liver microsomes (RLM, 250 μg mL^{-1}) and NADPH

(100 μM) in a hypoxic environment of glove box, and (b) the temporal profile of HDSF fluorescence at 705 nm in diagram a. Blank: PBS buffer containing RLM and NADPH.

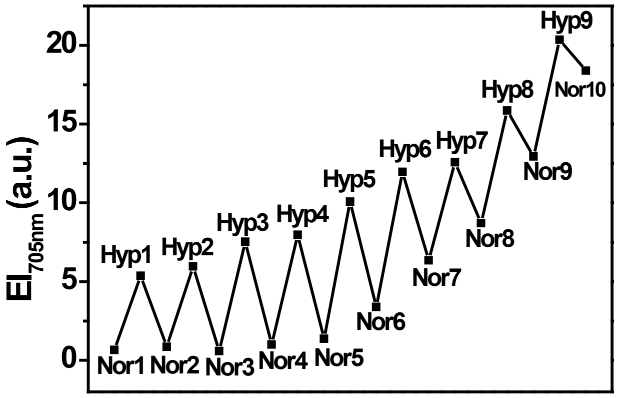

Supplementary Fig. 10 Fluorescence emission response of 20 μM HDSF at 705 nm in cycles of normoxia (in air until saturated by ambience) - hypoxia (in glove box, 15 min) incubation at 37 °C. RLM (250 μg mL^{-1}) and NADPH (100 μM) were added for each cycle.

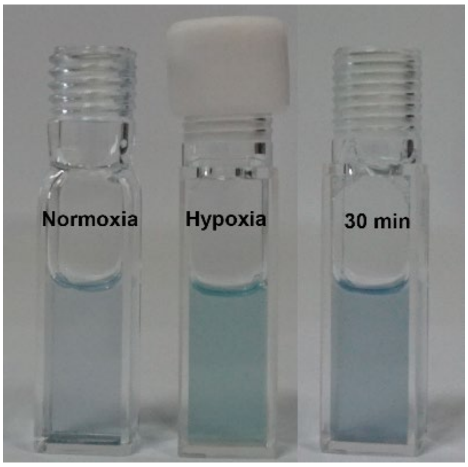

Supplementary Fig. 11 Images of 20 μM HDSF in PBS buffer containing RLM (250 μg mL^{-1}) and NADPH (100 μM) incubated at 37 °C in normoxia (in air)-hypoxia (in glove box, 15 min)-normoxia (30 min in air) cycle.

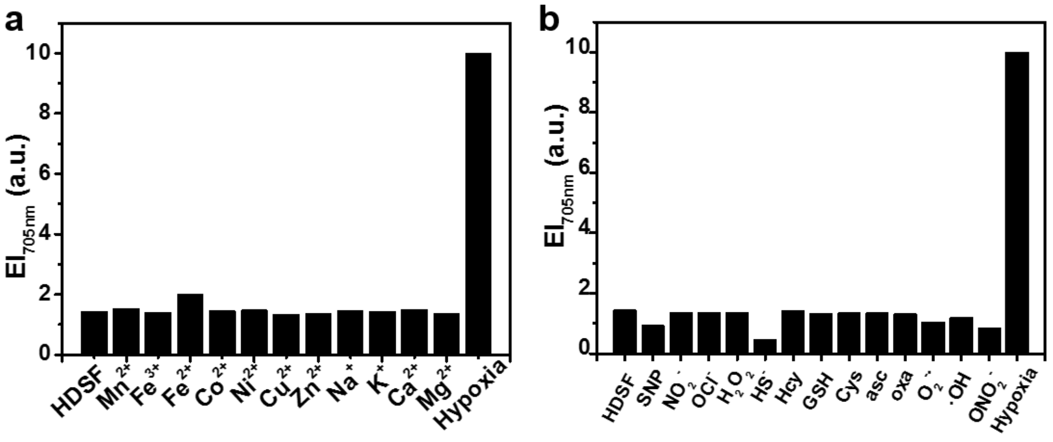

Supplementary Fig. 12 (a) Histogram of HDSF (20 μM in PBS buffer) fluorescence at 705 nm in the presence of 2 mM Na^+, K^+, Ca^{2+}, Mg^{2+}, 50 μM Mn^{2+}, Fe^{2+}, Fe^{3+}, Co^{2+}, Ni^{2+}, Cu^{2+}, and Zn^{2+}; (b) histogram of HDSF fluorescence at 705 nm in the presence of 100 μM sodium nitroferricyanide (Ⅲ) (SNP), NO_2^-, OCl^-, H_2O_2, HS^-, Hcy, GSH, Cys, ascorbic acid (asc), oxalic acid (oxa), •OH, 20 μM $O_2^{-•}$, and ONO_2^- respectively.

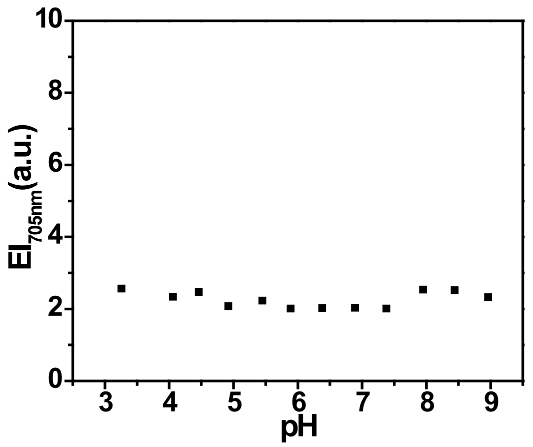

Supplementary Fig. 13 Fluorescence of HDSF (20 μM) at 705 nm in PBS buffer (10 mM) with different pH values. pH was adjusted by HCl and NaOH solutions.

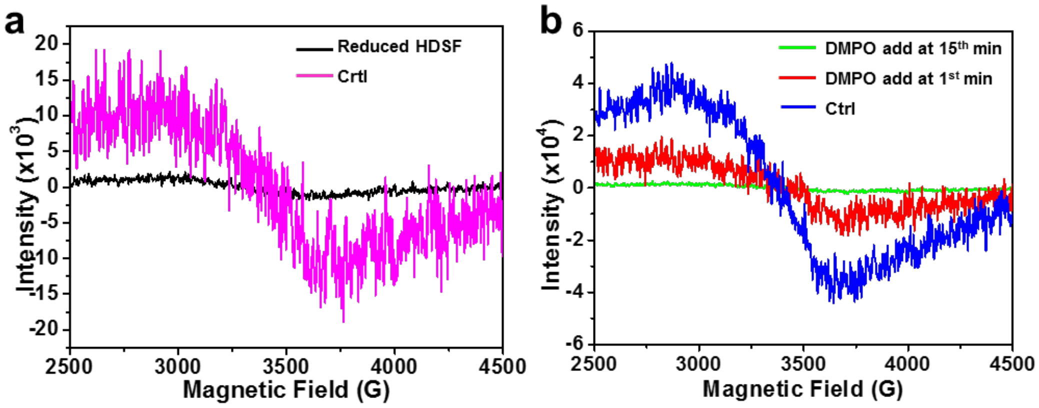

Supplementary Fig. 14 (a) EPR spectra of HDSF (1.5 mM) in PBS buffer (0.1 M, pH 7.4, 10% DMF, v/v) containing rat liver microsomes (RLM, 20 mg mL^{-1}) and NADPH (3 mM) incubated at 37 °C in hypoxia condition (glove box) for 15 min. Ctrl: PBS containing RLM and NADPH. (b) Radical capture agent 5,5-Dimethyl-1-pyrroline-N-oxide (DMPO) was added in the aforementioned solution in the beginning or at the end of reaction respectively. Signal of pure DMPO was collected as a control.

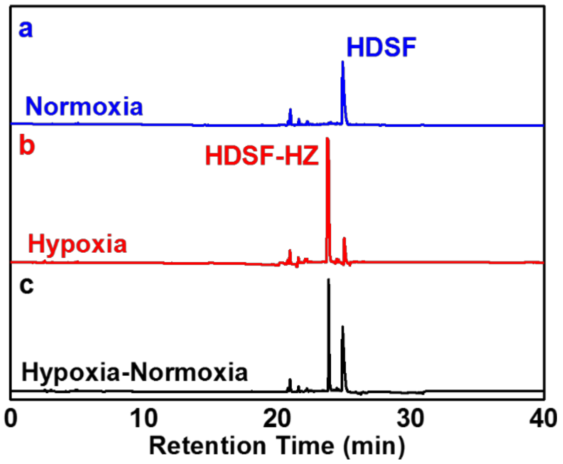

Supplementary Fig. 15 HPLC spectra of HDSF solution (20 μM in PBS buffer, pH 7.4) incubated with RLM (250 μg mL^{-1}) and NADPH (100 μM) at 37 °C for 20 min (a) in air, (b) in hypoxic condition (glove box), (c) solution of (b) exposed in air for 30 min. Elution solvent: mixture of methanol and water (1:1 v/v) containing 0.1% TFA. Data collected at 680 nm.

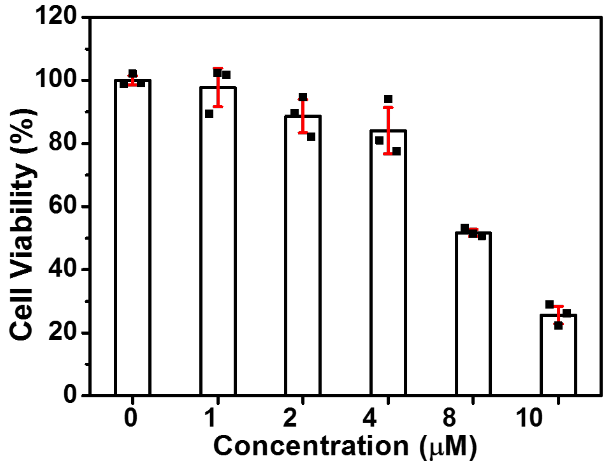

Supplementary Fig. 16 MCF-7 cell viability after treated with different concentrations of HDSF for 12 h, measured by MTT assay. Data are mean ± SD, n = 3 biologically independent experiments per group. Source data are available as a Source data file.

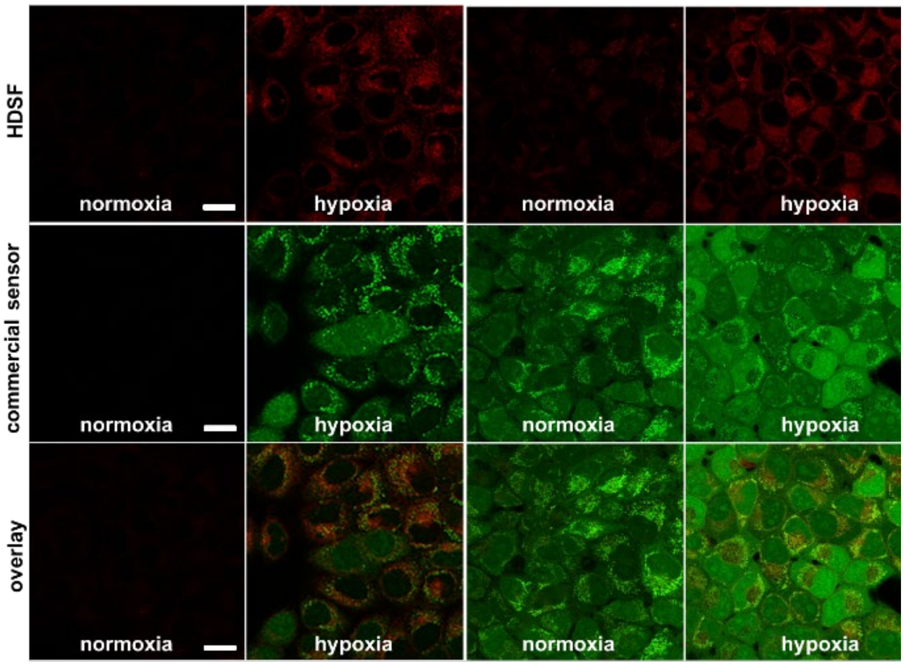

Supplementary Fig. 17 MCF-7 cells co-stained with HDSF and Image-iT^{TM} Green Hypoxia Reagent in hypoxia-normoxia cycles. Red channel was obtained with a band path of 640-750 nm upon excitation at 633 nm, and green channel was obtained with a band path of 492-630 nm upon excitation at 488 nm. The results are representative of three biologically independent experiments. Scale bars: 20 μm.

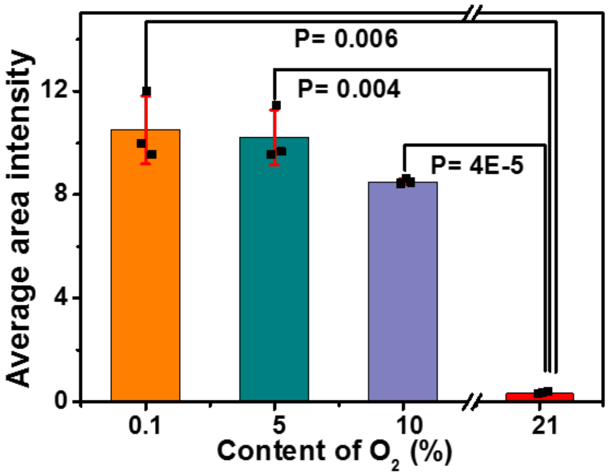

Supplementary Fig. 18 Histogram of average cell intensity in MCF-7 cells incubated in conditions with different O_2 contents. Data are presented as mean ± SD. Results are representative of three biologically independent experiments. Statistical significance was calculated with two-tailed Student's t test, when $p < 0.05$, it was considered to have statistical significance. Source data are available as a Source data file.

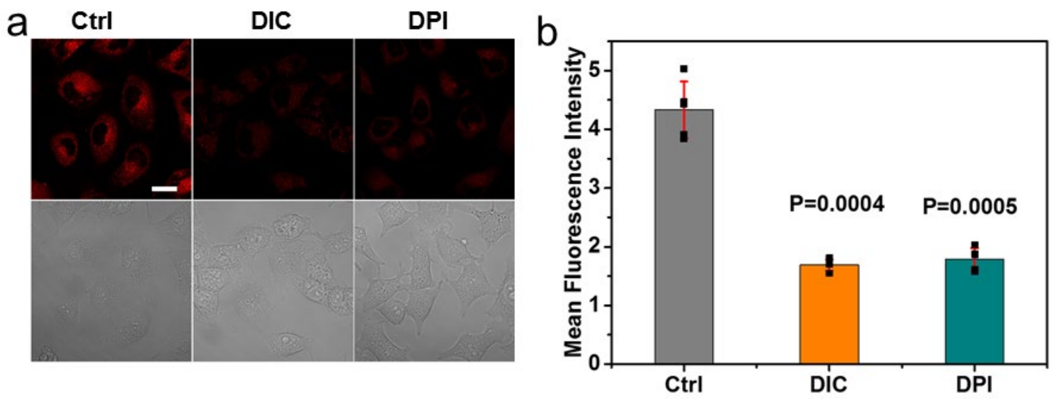

Supplementary Fig. 19 (a) Confocal images of MCF-7 cells treated under hypoxia condition for 4 h totally, that stained with HDSF (2 μM) for 1 h as a control (Ctrl), treated with dicoumarol (DIC, 20 μM) for 2 h and HDSF (2 μM) for 1 h, or treated with diphenyliodonium chloride (DPI, 100 μM) for 2 h and HDSF (2 μM) for 1 h respectively. Bottom: Corresponding bright field images. (b) Histogram of average fluorescence intensity in cells of diagram a, from n = 5 biologically independent experiments, mean ± SD. Statistical significance was calculated with two-tailed Student's t test, when $p < 0.05$, it was considered to have statistical significance. Scale bar: 20 μm. Source data are available as a Source data file.

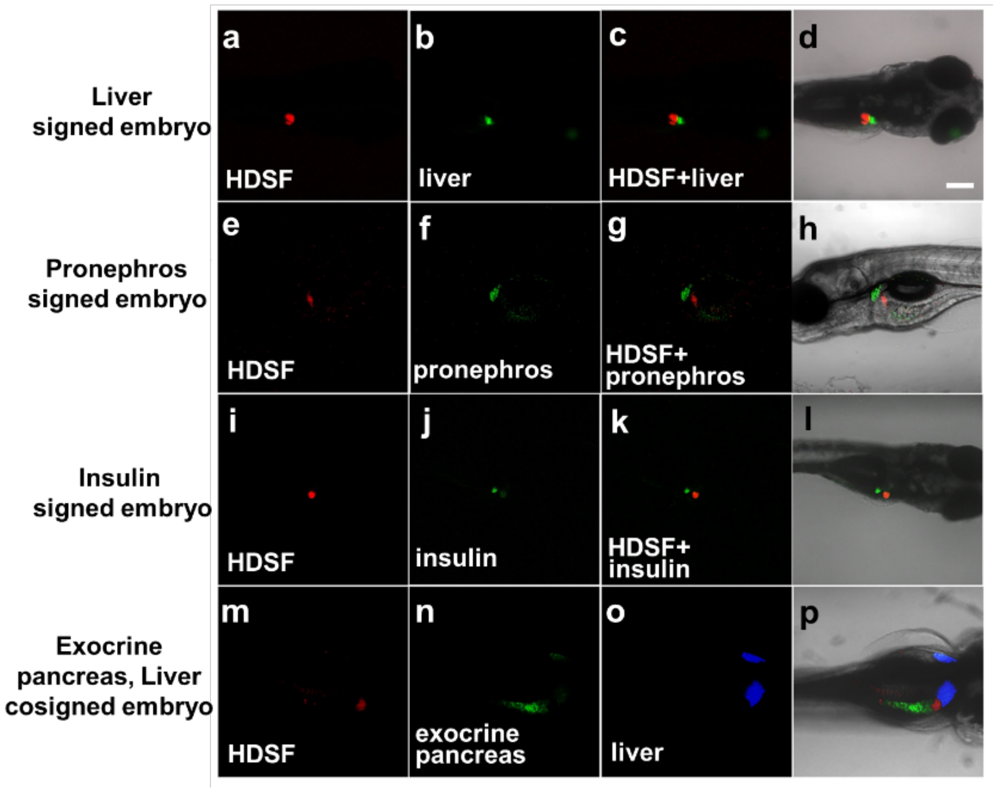

Supplementary Fig. 20 Confocal images of transgenic zebrafish embryos injected with HDSF (2 μM) and incubated with BDM (15 mM, 5 min) before imaging. Fluorescent protein (GFP or RFP) signed certain organs in embryos respectively, liver (a-d), pronephros (e-h), insulin (i-l), exocrine pancreas and liver (m-p). Pseudocolored images obtained with a band path of 640-750 nm upon excitation of HDSF at 633 nm (a, e, i, m), obtained with a band path of 500-600 nm upon excitation of GFP at 488 nm (b, f, j, n), or obtained with a band path of 550-700 nm

upon excitation of RFP at 543 nm (o). The results are representative of 5 biologically independent experiments. Scale bars: 200 μm.

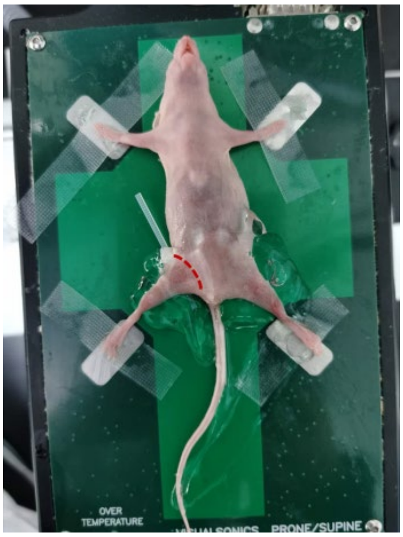

Supplementary Fig. 21 Picture of mouse during Doppler Ultrasound imaging. Blood flow of the right hind limb was imaged by Doppler Ultrasound before, during and after binding the right limb with a tourniquet (binding position is indicated by the red dash), and movies are supplied as additional Supplementary Movies.

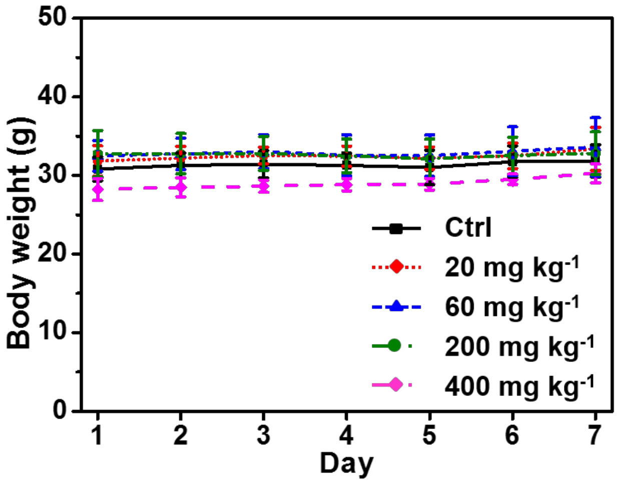

Supplementary Fig. 22 Body weight changes of mice over 7 days, after injected with 50 μL saline (control) or HDSF solution at the first day. Data are mean ± SD, n = 5 biologically independent mice per group.

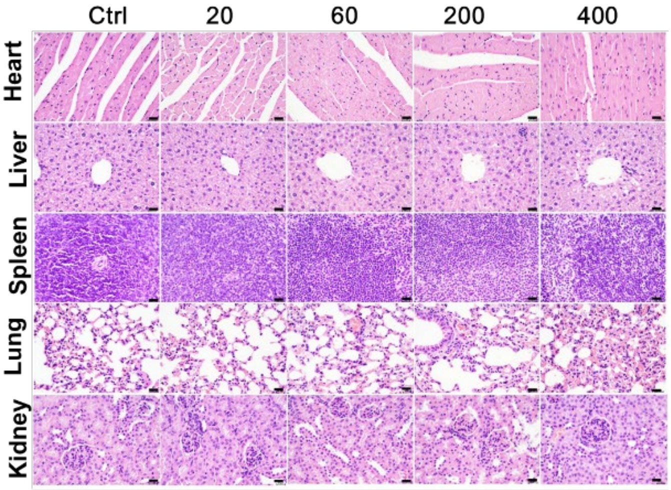

Supplementary Fig. 23 H&E staining of main organs of mice injected with saline (Ctrl) or HDSF solution (20 mg kg^{-1}, 60 mg kg^{-1}, 200 mg kg^{-1}, 400 mg kg^{-1}, respectively) at the first day and sacrificed after 7 days for H&E staining tests. Results are representative of 5 biologically independent experiments. Scale bars: 20 μm.

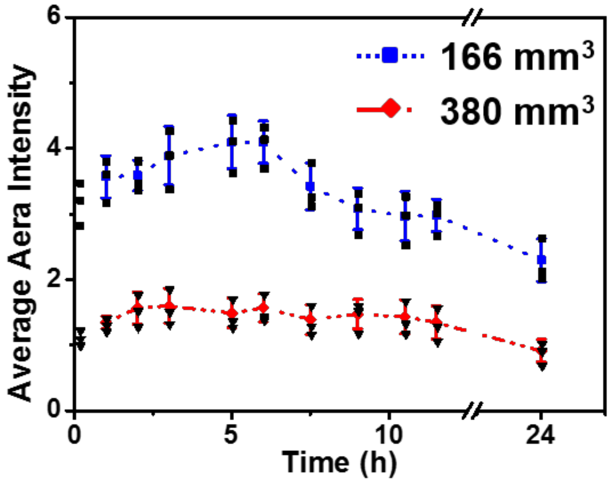

Supplementary Fig. 24 Temporal profile of average fluorescence intensity in tumour with different size after intratumorous HDSF-injection (20 μM, 50 μL). The results are presented as the mean ± SD, n = 3 biologically independent mice per group. λ_{ex}, 660 nm; λ_{em}, 710 nm.

3. Computational Details

DFT calculations were performed with Gaussian 09.[3] Geometry optimizations of all the ground state and transition structures were carried out at the B3LYP-D3 level of theory with the 6-31G(d) basis set in water using the CPCM model.[4-9] Vibrational frequencies were computed at the same level to verify that optimized structures are local minimums or transition states and to evaluate zero-point vibrational energies (ZPVE) and thermal corrections at 298 K. Solvent effects in water were evaluated at the more accurate B3LYP-D3/6-311+G(d,p) level with the CPCM model using the above optimized structures.

DFT-Computed Energies and Cartesian Coordinates

3,5-Bis(trifluoromethyl)aniline

G(H_2O) = -961.9464623 Hartree

C	-1.207205	1.419405	0.029480
C	-1.195536	0.027415	0.030933
C	1.195766	0.027214	-0.039550
C	1.207500	1.419062	-0.041964
C	0.000167	2.139588	-0.008236
H	-2.150595	1.954158	0.061203
H	2.150774	1.953949	-0.074438
C	0.000009	-0.690624	-0.003711
H	-0.000016	-1.772886	-0.000238
C	2.501245	-0.716172	-0.002080
C	-2.501417	-0.715749	0.004682
F	-2.410017	-1.925102	0.602052
F	-3.489571	-0.033571	0.626346
F	-2.923660	-0.941022	-1.264247
F	2.413762	-1.926284	-0.598521
F	2.913477	-0.940035	1.270418
F	3.494569	-0.035322	-0.616861
N	-0.000737	3.522691	-0.072134
H	-0.831621	3.978984	0.283909
H	0.846419	3.978155	0.244473

(3,5-bis(trifluoromethyl)phenyl)amino radical

G(H_2O) = -961.3011364 Hartree

C	-1.228561	1.470798	0.023400
C	-1.209405	0.088464	0.020903
C	1.212518	0.095719	-0.028686
C	1.221292	1.477862	-0.027291
C	-0.005135	2.214068	-0.001051
H	-2.170826	2.007141	0.046043
H	2.149971	2.035291	-0.048594
N	0.065704	3.551910	-0.001330
C	0.003735	-0.615350	-0.004901
H	0.006759	-1.698961	-0.007111
C	2.505320	-0.674962	-0.002206
C	-2.494250	-0.695949	0.002200
F	-2.446886	-1.739852	0.859869
F	-3.558718	0.059843	0.338497
F	-2.739133	-1.211026	-1.224801
F	2.443535	-1.773283	-0.787884
F	2.797141	-1.104974	1.247332
F	3.549240	0.068236	-0.421583
H	-0.885527	3.938767	0.021890

N1,N1-dimethylbenzene-1,4-diamine

G(H_2O) = -421.577365 Hartree

C	-0.092661	1.200362	-0.073227
C	-1.484547	1.197076	-0.016655
C	-2.212824	-0.000002	0.009173
C	-1.484551	-1.197082	-0.016620
C	-0.092664	-1.200375	-0.073190
C	0.646879	-0.000008	-0.117876
H	0.414045	2.157628	-0.078034
H	-2.014141	2.146692	0.014974
H	-2.014149	-2.146696	0.015038
H	0.414034	-2.157644	-0.077965
N	2.046919	0.000005	-0.220561
C	2.745226	-1.231721	0.115791
H	2.548487	-1.572525	1.146337
H	2.460283	-2.038128	-0.567434
H	3.819319	-1.071837	0.003497
C	2.745187	1.231735	0.115856
H	2.460239	2.038163	-0.567344
H	2.548411	1.572491	1.146410
H	3.819286	1.071885	0.003581
N	-3.623259	0.000001	-0.002780
H	-4.020850	-0.826506	0.432428
H	-4.020845	0.826529	0.432393

(4-(dimethylamino)phenyl)amino radical

G(H_2O) = -420.9515065 Hartree

C	-0.163285	1.211813	0.001609
C	-1.538262	1.200355	0.001645
C	-2.295467	-0.021704	-0.000087
C	-1.520635	-1.232146	-0.001785
C	-0.146962	-1.225678	-0.001797
C	0.585517	-0.001367	-0.000174
H	0.352468	2.164233	0.003583
H	-2.079757	2.143716	0.003288
H	-2.062049	-2.173786	-0.003301
H	0.382435	-2.170550	-0.003646

N	1.953506	0.008041	-0.000350
C	2.697427	-1.244733	0.003580
H	2.464619	-1.845197	0.891557
H	2.474853	-1.845380	-0.887005
H	3.764411	-1.025287	0.009889
C	2.680147	1.270869	-0.002967
H	2.438446	1.869297	-0.889944
H	2.450011	1.867296	0.888585
H	3.750016	1.066102	-0.010415
N	-3.627409	-0.100617	-0.000199
H	-4.009012	0.853132	0.001103

1-methyl-1,4-dihydropyridine-3-carboxamide

$G(H_2O)$ = -457.5293925 Hartree

C	0.374061	-0.785832	0.014292
C	-0.608064	0.153292	-0.012299
C	1.202478	1.855764	-0.107894
C	2.089739	0.853558	-0.106992
H	0.148822	-1.843898	0.093546
H	1.573457	2.873693	-0.179073
H	3.160104	1.016408	-0.168113
N	1.708922	-0.494519	-0.042200
C	2.719967	-1.518859	0.179742
H	2.294728	-2.498964	-0.044947
H	3.570779	-1.349032	-0.486369
H	3.079313	-1.518770	1.216749
C	-0.288694	1.638240	-0.017025
H	-0.701389	2.123274	0.882345
H	-0.803340	2.135955	-0.854176
C	-2.029970	-0.208887	0.029948
O	-2.899468	0.637952	0.279855
N	-2.379789	-1.533618	-0.171237
H	-3.375531	-1.677343	-0.287951
H	-1.812230	-2.091641	-0.795432

3-carbamoyl-1-methylpyridin-1-ium

$G(H_2O)$ = -456.7928178 Hartree

C	0.397551	-0.784222	0.069385
C	-0.604616	0.170374	-0.024033
C	1.105199	1.866212	-0.163435
C	2.063115	0.872869	-0.076691
H	0.198525	-1.840153	0.190659
H	1.420051	2.898158	-0.253687
H	3.126531	1.073160	-0.093502
N	1.698931	-0.424886	0.044012
C	2.760110	-1.453850	0.159631
H	2.294977	-2.434398	0.231457
H	3.392012	-1.405966	-0.727479
H	3.347378	-1.252319	1.055981
C	-0.241777	1.516132	-0.124348
H	-1.021509	2.267466	-0.169148
C	-2.072808	-0.174967	0.045286
O	-2.875175	0.667580	0.436027
N	-2.412135	-1.437477	-0.307391
H	-3.398893	-1.662548	-0.328466
H	-1.805896	-2.022785	-0.865144

3-carbamoyl-1-methylpyridinyl radical

$G(H_2O)$ = -456.9203753 Hartree

C	0.366006	-0.772726	0.039308
C	-0.612526	0.187544	-0.020460
C	1.139197	1.876675	-0.113647
C	2.097305	0.885951	-0.075116
H	0.141490	-1.827649	0.142904
H	1.476174	2.907903	-0.171762
H	3.164103	1.057415	-0.100787
N	1.694758	-0.465538	0.011034
C	2.720029	-1.490150	0.143842
H	2.252530	-2.475543	0.149698
H	3.419160	-1.433935	-0.697579
H	3.281498	-1.355311	1.075833
C	-0.225489	1.581362	-0.080318
H	-0.987271	2.348283	-0.104334
C	-2.047461	-0.163399	0.035788
O	-2.908135	0.678431	0.319813
N	-2.393698	-1.478839	-0.198528
H	-3.389298	-1.637331	-0.292785
H	-1.823100	-2.032191	-0.823616

$H_2PO_4^-$

$G(H_2O)$ = -643.858024 Hartree

P	0.017767	0.095925	0.128104
O	-0.249391	-0.720678	1.367293
O	0.366517	1.555742	0.161278

O	1.220464	-0.645295	-0.745343
H	1.195489	-1.598290	-0.561263
O	-1.294407	-0.065707	-0.880686
H	-1.807447	-0.833082	-0.580628

HPO_4^{2-}

$G(H_2O)$ = -643.3834211 Hartree

P	-0.135188	-0.001689	0.064859
O	-0.880664	1.297054	-0.282367
O	0.485032	-0.038977	1.475828
O	-0.875588	-1.283436	-0.350622
O	1.276781	0.027729	-0.930958
H	1.983326	0.006376	-0.267930

HD-NH

$G(H_2O)$ = -1269.76921 Hartree

C	-5.276630	-2.785200	0.215053
C	-3.996298	-2.106766	0.160896
C	-6.457525	-1.923758	0.142428
C	-3.933738	-0.749024	0.046107
H	-3.078425	-2.685580	0.212167
C	-6.370645	-0.573007	0.029286
H	-7.422612	-2.420327	0.183512
C	-5.098711	0.090240	-0.026496
H	-7.267423	0.039197	-0.022548
C	-4.917396	1.457219	-0.139101
C	-2.527753	1.193929	-0.120164
C	-3.632941	2.031959	-0.189201
H	-5.788203	2.106917	-0.188706
C	-1.165350	1.634567	-0.175518
O	-2.694890	-0.158856	0.001399
C	-3.425356	3.522396	-0.318496
H	-3.444192	3.811193	-1.379934
H	-4.258806	4.049856	0.159518
C	-2.090315	3.945728	0.299525
H	-2.129693	3.786908	1.384720
H	-1.916109	5.013836	0.132419
C	-0.934148	3.129236	-0.290149
H	-0.002145	3.399802	0.216700
H	-0.800515	3.403856	-1.347307
C	-0.140748	0.715797	-0.155684
H	-0.433539	-0.322751	-0.078662
C	1.244312	1.010403	-0.243729
H	1.527227	2.051951	-0.345773
C	2.257176	0.081155	-0.218510
C	4.417019	-0.707078	-0.215496
C	3.624414	-1.849035	-0.052394
C	5.808186	-0.773240	-0.236736
C	4.216793	-3.094574	0.077461
C	6.395923	-2.037829	-0.102561
H	6.423291	0.113227	-0.345827
C	5.617221	-3.187499	0.050388
H	3.609700	-3.987287	0.201003
H	7.478869	-2.118921	-0.115534
H	6.096098	-4.156289	0.153191
N	3.587357	0.416725	-0.342555
C	4.099313	1.773312	-0.478730
H	5.056278	1.715464	-1.004706
H	3.420682	2.333956	-1.128419
C	4.270210	2.479101	0.871130
H	3.304378	2.491508	1.389955
H	4.955542	1.889193	1.491871
C	4.799480	3.903568	0.701675
H	4.922418	4.393849	1.672730
H	5.773383	3.905744	0.197888
H	4.111192	4.511503	0.102689
C	2.158577	-1.445987	-0.048050
C	1.510983	-1.831655	1.299542
H	2.034508	-1.349640	2.130680
H	0.458367	-1.541774	1.341285
H	1.572167	-2.916034	1.437908
C	1.420980	-2.108614	-1.232272
H	0.366631	-1.822884	-1.263118
H	1.882743	-1.825132	-2.182875
H	1.477002	-3.197629	-1.134465
N	-5.454638	-4.079244	0.321983
H	-4.536949	-4.537302	0.361218

$HD-NH_2$

$G(H_2O)$ = -1270.245614 Hartree

C	-5.226469	-2.672696	0.247574
C	-3.965802	-2.051585	0.185671
C	-6.400597	-1.870207	0.171874
C	-3.905215	-0.676212	0.049020
H	-3.049548	-2.629031	0.243852

C	-6.311200	-0.503729	0.036892
H	-7.371303	-2.353879	0.220506
C	-5.054600	0.141701	-0.030701
H	-7.212514	0.099069	-0.019151
C	-4.862519	1.539329	-0.174189
C	-2.475221	1.230779	-0.139056
C	-3.604400	2.089979	-0.238344
H	-5.735014	2.184023	-0.240165
C	-1.139099	1.672583	-0.177837
O	-2.664719	-0.108906	-0.000200
C	-3.359522	3.567535	-0.424967
H	-3.292335	3.789652	-1.499550
H	-4.214871	4.131443	-0.039266
C	-2.058872	3.994590	0.260288
H	-2.157513	3.854556	1.343943
H	-1.870806	5.058216	0.084355
C	-0.878417	3.164637	-0.255775
H	0.016472	3.408084	0.326619
H	-0.656098	3.447354	-1.294956
C	-0.104540	0.735615	-0.162607
H	-0.404773	-0.301688	-0.104828
C	1.258863	1.029289	-0.232251
H	1.552160	2.069456	-0.305003
C	2.275670	0.078608	-0.216859
C	4.420519	-0.725466	-0.216652
C	3.619728	-1.862408	-0.085294
C	5.810059	-0.789589	-0.236010
C	4.205534	-3.114558	0.017631
C	6.391225	-2.058590	-0.128687
H	6.427859	0.096872	-0.323876
C	5.604530	-3.207656	-0.005772
H	3.596258	-4.008169	0.116829
H	7.473238	-2.145634	-0.139662
H	6.080041	-4.179925	0.075791
N	3.587885	0.409490	-0.316103
C	4.116366	1.768569	-0.425325
H	5.070425	1.705374	-0.953701
H	3.443966	2.348804	-1.061778
C	4.295735	2.433860	0.943276
H	3.328484	2.453736	1.459041
H	4.966985	1.815926	1.551332
C	4.854078	3.850764	0.806965
H	4.981073	4.314180	1.790325
H	5.830421	3.844249	0.308589
H	4.180622	4.485545	0.219501
C	2.159319	-1.447641	-0.078466
C	1.503399	-1.850048	1.261556
H	2.026937	-1.387038	2.103035
H	0.452287	-1.555821	1.303902
H	1.558433	-2.936735	1.377755
C	1.420179	-2.076942	-1.280981
H	0.366086	-1.791005	-1.304235
H	1.882538	-1.771620	-2.224102
H	1.476252	-3.167219	-1.206892
N	-5.333621	-4.032810	0.332883
H	-4.522736	-4.563140	0.621531
H	-6.217515	-4.424213	0.628562

HDMA

G(H_2O) = -1689.379139 Hartree

C	-6.940485	-2.192969	0.130453
C	-5.936480	-1.252016	0.086624
C	-6.237334	0.129345	0.046142
C	-7.592144	0.516897	0.051553
C	-8.609725	-0.414837	0.094982
C	-8.315096	-1.805887	0.135808
H	-6.674648	-3.241917	0.160447
H	-4.897222	-1.560348	0.082721
H	-7.821319	1.577836	0.020883
H	-9.636610	-0.073799	0.098423
N	-9.307552	-2.738654	0.178430
C	-3.172564	1.869487	-0.047738
C	-1.818886	1.511455	-0.054880
C	-3.539225	3.238551	-0.083336
C	-0.858806	2.511521	-0.096011
H	-1.531407	0.466795	-0.026341
C	-2.568848	4.217203	-0.125869
H	-4.591593	3.492621	-0.076602
C	-1.196238	3.878572	-0.133859
H	-2.846786	5.266598	-0.155072
C	-0.128106	4.823062	-0.183768
C	1.475214	3.019805	-0.136602
C	1.179534	4.419073	-0.196436
H	-0.365875	5.882536	-0.220248
C	2.773971	2.493650	-0.123327
O	0.452255	2.126549	-0.093164
C	2.337981	5.379940	-0.288698

H	2.595359	5.527010	-1.347352
H	2.034882	6.356267	0.102019
C	3.558985	4.833820	0.455687
H	3.326838	4.755772	1.524985
H	4.402008	5.523872	0.353968
C	3.947017	3.453913	-0.085131
H	4.740467	3.031261	0.540239
H	4.371893	3.558524	-1.093787
C	2.951853	1.103659	-0.160432
H	2.047857	0.510785	-0.181993
C	4.178295	0.448171	-0.185635
H	5.079132	1.049099	-0.176213
C	-10.704603	-2.322177	0.177821
H	-10.937066	-1.702598	1.052603
H	-10.949370	-1.750470	-0.725572
H	-11.339699	-3.206505	0.205696
C	-8.985172	-4.160003	0.227642
H	-8.420952	-4.473343	-0.659099
H	-8.394816	-4.405071	1.118758
H	-9.910848	-4.733001	0.263379
C	4.333605	-0.939779	-0.221752
C	5.438737	-2.943558	-0.238278
C	4.085912	-3.289274	-0.216417
C	6.454408	-3.894022	-0.232485
C	3.712824	-4.624461	-0.201656
C	6.066684	-5.238657	-0.215294
H	7.503429	-3.620597	-0.235157
C	4.716800	-5.602962	-0.202575
H	2.665408	-4.911086	-0.186785
H	6.832094	-6.008267	-0.209920
H	4.443071	-6.653152	-0.189659
N	5.546963	-1.534780	-0.263583
C	6.837416	-0.843645	-0.244832
H	7.556588	-1.484753	-0.759324
H	6.747947	0.067662	-0.840210
C	7.303174	-0.533906	1.181339
H	6.547644	0.088063	1.675600
H	7.360498	-1.472110	1.745677
C	8.658278	0.174340	1.185524
H	8.979255	0.389270	2.209511
H	9.429749	-0.444659	0.713308
H	8.608894	1.124568	0.641206
C	3.246823	-2.025016	-0.206231
C	2.401104	-1.964692	1.086293
H	3.043547	-1.990705	1.971186
H	1.788567	-1.061488	1.129716
H	1.734187	-2.831323	1.119186
C	2.358787	-1.962335	-1.469815
H	1.751489	-1.054741	-1.494842
H	2.970700	-1.995176	-2.375804
H	1.684706	-2.824065	-1.477852
N	-5.305788	1.154488	0.001460
N	-4.080509	0.796750	-0.002771

HDMA-HZ

G(H_2O) = -1690.576454 Hartree

C	6.253861	-1.379248	1.026451
C	5.523290	-0.703522	0.050807
C	6.056883	-0.491480	-1.223716
C	7.343242	-0.971612	-1.493132
C	8.077775	-1.640595	-0.520211
C	7.547909	-1.878201	0.768120
H	5.799453	-1.512952	2.000036
H	4.534414	-0.332692	0.298380
H	7.783109	-0.814387	-2.475170
H	9.070771	-1.986830	-0.777559
N	8.264234	-2.591224	1.730904
C	3.273541	1.221464	-1.560914
C	1.920599	1.028536	-1.234885
C	3.891403	2.470562	-1.293833
C	1.220089	2.074542	-0.660902
H	1.421880	0.083808	-1.424113
C	3.168681	3.493280	-0.718767
H	4.936249	2.594760	-1.546876
C	1.806343	3.328964	-0.382418
H	3.646137	4.446130	-0.511060
C	0.988891	4.320924	0.220991
C	-0.878891	2.800357	0.221891
C	-0.326261	4.075634	0.531643
H	1.422368	5.291725	0.446304
C	-2.210459	2.432006	0.485640
O	-0.093204	1.855306	-0.360464
C	-1.216864	5.089681	1.206760
H	-1.145518	4.963477	2.296485
H	-0.857382	6.098864	0.981855
C	-2.673610	4.908743	0.773078
H	-2.760470	5.116156	-0.300734

H	-3.315344	5.623914	1.296748
C	-3.147426	3.479240	1.056606
H	-4.148116	3.339666	0.633999
H	-3.248306	3.333087	2.141720
C	-2.623268	1.120631	0.238262
H	-1.874868	0.455702	-0.170590
C	-3.897519	0.610894	0.491113
H	-4.638800	1.279588	0.911179
C	9.694484	-2.769367	1.528730
H	9.890265	-3.351389	0.622190
H	10.238088	-1.814120	1.445805
H	10.103884	-3.327905	2.372098
C	7.805351	-2.529883	3.110257
H	7.787822	-1.503622	3.512499
H	6.797193	-2.947144	3.202525
H	8.469364	-3.133448	3.731312
C	-4.287061	-0.703756	0.245516
C	-5.691403	-2.510019	0.139995
C	-4.485262	-2.971556	-0.392118
C	-6.822258	-3.314783	0.233795
C	-4.380488	-4.279429	-0.839411
C	-6.706034	-4.633026	-0.222347
H	-7.760325	-2.948543	0.635170
C	-5.504005	-5.113667	-0.750157
H	-3.449096	-4.653597	-1.254261
H	-7.569030	-5.288983	-0.164573
H	-5.440283	-6.140119	-1.097084
N	-5.531308	-1.162486	0.529108
C	-6.627016	-0.364474	1.078821
H	-7.287287	-1.045553	1.620382
H	-6.213463	0.324576	1.818910
C	-7.396660	0.386942	-0.012141
H	-6.700294	1.036536	-0.555380
H	-7.782180	-0.340751	-0.736077
C	-8.542225	1.211028	0.576247
H	-9.084447	1.740519	-0.213386
H	-9.257826	0.571513	1.105834
H	-8.167713	1.957183	1.286691
C	-3.465105	-1.847705	-0.368868
C	-3.012816	-1.515460	-1.808741
H	-3.866355	-1.216678	-2.424149
H	-2.274184	-0.710922	-1.827238
H	-2.559421	-2.404555	-2.257058
C	-2.265585	-2.235610	0.525323

H	-1.517664	-1.440643	0.568696
H	-2.596792	-2.455980	1.544198
H	-1.787800	-3.131411	0.117225
N	5.360955	0.252915	-2.216268
H	5.683913	0.050280	-3.157077
N	3.967218	0.198513	-2.171807
H	3.563248	-0.731948	-2.082615

HDSF

G(H_2O) = -2229.730198 Hartree

C	-2.332490	2.817958	0.014663
C	-1.041998	2.270121	0.002831
C	-2.518867	4.213533	0.009521
C	0.035210	3.137467	-0.012991
H	-0.894164	1.197671	0.005320
C	-1.426380	5.064176	-0.005180
H	-3.532543	4.598252	0.017509
C	-0.119738	4.541048	-0.018314
H	-1.563020	6.140598	-0.010067
C	1.068219	5.339954	-0.048317
C	2.417179	3.334626	-0.051429
C	2.306723	4.767126	-0.077362
H	0.970083	6.421520	-0.059906
C	3.631490	2.647904	-0.064031
O	1.284206	2.583186	-0.020743
C	3.581650	5.567220	-0.162154
H	3.840867	5.703886	-1.221746
H	3.415119	6.565272	0.254340
C	4.730038	4.846864	0.548626
H	4.507148	4.775415	1.620236
H	5.655086	5.421600	0.444849
C	4.922588	3.440881	-0.028163
H	5.661513	2.901154	0.573340
H	5.340695	3.509795	-1.042610
C	3.624363	1.241435	-0.126664
H	2.650587	0.772241	-0.131486
C	4.750011	0.437474	-0.200274
H	5.722177	0.914285	-0.214783
C	4.718737	-0.963101	-0.258973
C	5.549347	-3.091270	-0.343565
C	4.166962	-3.258553	-0.246456
C	6.433885	-4.162965	-0.399278
C	3.625621	-4.534718	-0.213668

C	5.877371	-5.446132	-0.362633
H	7.507094	-4.025155	-0.462709
C	4.494182	-5.633205	-0.273029
H	2.552648	-4.684610	-0.140876
H	6.535422	-6.308258	-0.403000
H	4.088790	-6.639575	-0.246436
N	5.837134	-1.706659	-0.368791
C	7.208281	-1.193004	-0.424867
H	7.797437	-1.908276	-1.002851
H	7.202448	-0.259801	-0.991305
C	7.803811	-0.994902	0.972651
H	7.173010	-0.294349	1.532375
H	7.772349	-1.949978	1.509714
C	9.239127	-0.473098	0.895778
H	9.653857	-0.333676	1.898850
H	9.886338	-1.174785	0.357256
H	9.280662	0.491053	0.376072
C	3.501626	-1.896275	-0.190727
C	2.735941	-1.733978	1.142373
H	3.411466	-1.851010	1.994509
H	2.251488	-0.757850	1.215790
H	1.962370	-2.504716	1.207222
C	2.566853	-1.711086	-1.408822
H	2.067364	-0.739768	-1.395501
H	3.125195	-1.802210	-2.344806
H	1.798347	-2.489273	-1.389023
N	-3.334725	0.801533	0.067252
N	-3.516096	2.049652	0.027127
C	-4.524708	0.036132	0.066969
C	-4.350861	-1.345784	0.179157
C	-5.815796	0.575838	-0.044741
C	-5.462806	-2.187366	0.188687
H	-3.344919	-1.742028	0.255749
C	-6.912371	-0.275144	-0.038085
H	-5.936002	1.647796	-0.133112
C	-6.747317	-1.660143	0.080582
C	-5.270375	-3.667629	0.374584
C	-8.300214	0.279304	-0.216275
H	-7.610376	-2.314978	0.086170
F	-6.283059	-4.379714	-0.163774
F	-4.122315	-4.097073	-0.192728
F	-5.207803	-3.999121	1.685694
F	-9.198778	-0.373030	0.554450
F	-8.721946	0.154868	-1.496657
F	-8.368115	1.589440	0.098038

HDSF-HZ

G(H_2O) = -2230.942742 Hartree

C	5.499283	-0.843318	1.197103
C	4.689011	-0.431932	0.138708
C	5.231934	-0.319537	-1.146919
C	6.585193	-0.631662	-1.351876
C	7.371871	-1.041589	-0.281605
H	3.647124	-0.198877	0.316289
H	7.014815	-0.550244	-2.344661
C	2.377800	1.146589	-1.638496
C	1.028256	0.961074	-1.303524
C	2.981077	2.417013	-1.470194
C	0.312578	2.041024	-0.815404
H	0.541484	-0.001428	-1.419981
C	2.243498	3.473489	-0.977177
H	4.024780	2.538754	-1.730345
C	0.883608	3.318531	-0.632250
H	2.709326	4.444999	-0.843390
C	0.051699	4.344888	-0.104951
C	-1.799867	2.806494	-0.002486
C	-1.260225	4.108136	0.218358
H	0.474078	5.334168	0.049440
C	-3.130074	2.447322	0.270801
O	-0.999075	1.829096	-0.505872
C	-2.165041	5.158021	0.815335
H	-2.096455	5.109984	1.911456
H	-1.814946	6.152366	0.520534
C	-3.618479	4.931547	0.391113
H	-3.704439	5.063647	-0.694578
H	-4.269041	5.674238	0.862959
C	-4.077965	3.520088	0.771137
H	-5.077151	3.342259	0.359887
H	-4.176413	3.446312	1.863731
C	-3.535182	1.121194	0.088817
H	-2.780205	0.440717	-0.280057
C	-4.808064	0.618924	0.354069
H	-5.555546	1.301117	0.740039
C	-5.191942	-0.706909	0.156413
C	-6.591760	-2.518206	0.107686
C	-5.383701	-2.994489	-0.406431
C	-7.721912	-3.320603	0.225378

C	-5.276199	-4.316061	-0.811040
C	-7.602940	-4.652620	-0.187711
H	-8.661199	-2.942613	0.612711
C	-6.399011	-5.148138	-0.697345
H	-4.343683	-4.702475	-1.211766
H	-8.465102	-5.307650	-0.110784
H	-6.333359	-6.185053	-1.011178
N	-6.434613	-1.157819	0.452288
C	-7.533315	-0.344282	0.973530
H	-8.192486	-1.008628	1.536701
H	-7.122011	0.370285	1.690205
C	-8.302778	0.367559	-0.143846
H	-7.607077	0.999911	-0.707896
H	-8.685361	-0.385442	-0.842927
C	-9.450980	1.208527	0.414865
H	-9.991611	1.711749	-0.392802
H	-10.1671490.585353		0.962742
H	-9.078996	1.977415	1.101985
C	-4.365559	-1.868578	-0.416712
C	-3.908752	-1.582326	-1.865090
H	-4.761605	-1.313150	-2.494895
H	-3.177635	-0.771867	-1.907681
H	-3.444853	-2.482356	-2.279574
C	-3.168180	-2.224992	0.493734
H	-2.418736	-1.430425	0.509330
H	-3.501296	-2.407784	1.519366
H	-2.691332	-3.135611	0.118959
N	4.471063	0.148223	-2.222451
H	4.828454	-0.070524	-3.144627
N	3.088894	0.078995	-2.158576
H	2.697472	-0.847157	-2.006497
C	6.843299	-1.151225	1.006805
H	7.463160	-1.469079	1.835147
C	8.800625	-1.442846	-0.523598
C	4.911066	-0.884190	2.580279
F	4.932025	0.338090	3.165129
F	3.619316	-1.284694	2.566930
F	5.585498	-1.721124	3.397700
F	8.915031	-2.777875	-0.725375
F	9.593017	-1.140271	0.528849
F	9.323888	-0.836681	-1.611515

Int1-HDMA

G(H_2O) = -1690.10443 Hartree

C	6.668277	-1.011072	1.155502
C	5.726979	-0.857638	0.136147
C	6.062765	-1.104644	-1.200207
C	7.377073	-1.516612	-1.472094
C	8.315524	-1.674159	-0.458765
C	7.985608	-1.434488	0.893687
H	6.353483	-0.798282	2.169953
H	4.722645	-0.544322	0.393290
H	7.673308	-1.709170	-2.501213
H	9.314451	-1.990837	-0.733057
N	8.918384	-1.634293	1.924079
C	3.274308	0.222922	-1.610014
C	1.870358	0.272924	-1.277422
C	4.024204	1.465296	-1.468390
C	1.307523	1.430899	-0.831659
H	1.274413	-0.627975	-1.375561
C	3.433102	2.610283	-1.029722
H	5.072483	1.453526	-1.738508
C	2.045416	2.654393	-0.678826
H	4.010746	3.526953	-0.939130
C	1.371976	3.777575	-0.226982
C	-0.681906	2.536998	-0.061287
C	0.002169	3.738530	0.088683
H	1.913645	4.714016	-0.114962
C	-2.067301	2.347135	0.244710
O	-0.031368	1.424258	-0.519039
C	-0.740561	4.954780	0.589368
H	-0.633393	5.034073	1.681397
H	-0.286120	5.858813	0.168154
C	-2.226318	4.876496	0.229534
H	-2.336227	4.937876	-0.860662
H	-2.766975	5.725733	0.660303
C	-2.837534	3.561411	0.727252
H	-3.878448	3.494724	0.394039
H	-2.866625	3.570142	1.827198
C	-2.634573	1.097356	0.129049
H	-1.982378	0.308476	-0.220090
C	-3.974567	0.759590	0.444439
H	-4.613136	1.549896	0.822338
C	10.326286	-1.677333	1.556909
H	10.529638	-2.525042	0.894967
H	10.669133	-0.759390	1.050302
H	10.923585	-1.817491	2.460011

C	8.624561	-1.037590	3.218278
H	8.501113	0.057748	3.171468
H	7.708890	-1.462568	3.641928
H	9.440819	-1.264651	3.906807
C	-4.525890	-0.494807	0.321308
C	-6.154091	-2.115960	0.393640
C	-5.032732	-2.765633	-0.134530
C	-7.369932	-2.771919	0.571200
C	-5.104691	-4.104106	-0.484720
C	-7.431164	-4.124560	0.211843
H	-8.243808	-2.265228	0.965756
C	-6.317308	-4.788580	-0.307659
H	-4.238772	-4.618468	-0.892617
H	-8.366748	-4.661131	0.339298
H	-6.389990	-5.837014	-0.579551
N	-5.822262	-0.783659	0.682092
C	-6.778879	0.178874	1.210741
H	-7.494175	-0.369852	1.829776
H	-6.246515	0.860545	1.880662
C	-7.507441	0.955816	0.108728
H	-6.765104	1.461386	-0.520213
H	-8.035131	0.242399	-0.535881
C	-8.489385	1.973525	0.690567
H	-9.007447	2.517221	-0.105941
H	-9.248076	1.481334	1.310409
H	-7.970157	2.708486	1.316890
C	-3.877053	-1.781979	-0.220749
C	-3.429242	-1.622794	-1.689834
H	-4.260822	-1.276905	-2.311145
H	-2.606722	-0.910343	-1.788196
H	-3.090856	-2.589650	-2.076235
C	-2.709391	-2.256541	0.672216
H	-1.866949	-1.561013	0.642902
H	-3.035236	-2.359618	1.711625
H	-2.357458	-3.233112	0.324246
N	5.170144	-0.898302	-2.282746
H	5.342154	-1.594747	-3.001899
N	3.766689	-0.936162	-2.003742

Int1-HDSF

G(H_2O) = -2230.476022 Hartree

C	2.388647	0.352569	-1.592714
C	0.981780	0.360964	-1.285092
C	3.075521	1.635088	-1.554874
C	0.357678	1.526446	-0.947365
H	0.430691	-0.572717	-1.309987
C	2.423918	2.784810	-1.220753
H	4.127054	1.655543	-1.812378
C	1.033941	2.789826	-0.889257
H	2.957774	3.731619	-1.201249
C	0.301064	3.913391	-0.529533
C	-1.690793	2.589559	-0.283888
C	-1.063326	3.833633	-0.218522
H	0.797647	4.880019	-0.487567
C	-3.067635	2.360322	0.021206
O	-0.982760	1.480858	-0.651961
C	-1.867005	5.045729	0.189854
H	-1.770878	5.206613	1.273785
H	-1.452557	5.937821	-0.293163
C	-3.345159	4.871114	-0.167282
H	-3.452890	4.850375	-1.259188
H	-3.927531	5.721717	0.201802
C	-3.895369	3.566599	0.421014
H	-4.930807	3.428333	0.093409
H	-3.927298	3.649181	1.517722
C	-3.582070	1.081488	-0.029715
H	-2.895248	0.303095	-0.333119
C	-4.907401	0.706774	0.293954
H	-5.577668	1.485594	0.638714
C	-5.411299	-0.572904	0.213588
C	-6.979218	-2.248114	0.333008
C	-5.834613	-2.872456	-0.174333
C	-8.171904	-2.939718	0.528831
C	-5.858977	-4.222387	-0.485392
C	-8.185258	-4.303476	0.209097
H	-9.063498	-2.452083	0.907294
C	-7.047534	-4.942731	-0.289703
H	-4.975047	-4.717917	-0.877412
H	-9.101690	-4.868575	0.351022
H	-7.083132	-6.000490	-0.531068
N	-6.694721	-0.895717	0.581322
C	-7.685409	0.046540	1.085328
H	-8.379834	-0.510944	1.719892
H	-7.176756	0.764143	1.735669
C	-8.441758	0.766188	-0.036531
H	-7.719144	1.285901	-0.676815
H	-8.938918	0.016477	-0.663930

C -9.465284 1.756846 0.519720
H -10.0005212.262427 -0.290364
H -10.2068361.248479 1.147143
H -8.978117 2.524894 1.131973
C -4.714743 -1.851117 -0.288059
C -4.269625 -1.719087 -1.760749
H -5.112575 -1.426079 -2.393760
H -3.476158 -0.977505 -1.879484
H -3.891880 -2.683200 -2.116146
C -3.533081 -2.256769 0.620619
H -2.714541 -1.534402 0.570363
H -3.857143 -2.338953 1.662382
H -3.147732 -3.230901 0.302828
N 4.345529 -0.736461 -2.120066
H 4.590613 -1.371683 -2.871890
N 2.943651 -0.819216 -1.865248
C 5.239788 -0.789260 -1.061465
C 4.866408 -0.450179 0.251394
C 6.583271 -1.139246 -1.307729
C 5.819023 -0.450539 1.269369
H 3.839398 -0.192917 0.469630
C 7.512409 -1.133009 -0.275488
H 6.888701 -1.413955 -2.312282
C 7.149513 -0.785871 1.029806
C 5.371600 -0.148517 2.671245
C 8.951572 -1.440107 -0.575375
H 7.879889 -0.781989 1.828156
F 6.383202 0.301872 3.447049
F 4.394710 0.787366 2.703078
F 4.869412 -1.248817 3.286530
F 9.573515 -2.028328 0.472430
F 9.655741 -0.315599 -0.859325
F 9.091804 -2.263095 -1.639056

Int2-HDMA

G(H_2O) = -1690.707274 Hartree

C 6.100143 -1.450262 1.002119
C 5.443926 -0.765976 -0.020339
C 6.070047 -0.542627 -1.250970
C 7.374153 -1.023107 -1.423684
C 8.032524 -1.702681 -0.404781
C 7.408169 -1.950450 0.838326
H 5.572749 -1.589098 1.937715
H 4.441171 -0.393508 0.158392
H 7.887236 -0.858853 -2.368571
H 9.041625 -2.048558 -0.591441
N 8.050029 -2.675753 1.846728
C 3.381072 1.225416 -1.667230
C 2.030555 1.037598 -1.323907
C 4.003093 2.451061 -1.379177
C 1.329655 2.067102 -0.714634
H 1.525185 0.098726 -1.530461
C 3.279849 3.467373 -0.764082
H 5.046123 2.585347 -1.637619
C 1.927188 3.311441 -0.414902
H 3.768314 4.412309 -0.540195
C 1.116063 4.310794 0.224322
C -0.767315 2.788238 0.218508
C -0.197928 4.042937 0.538543
H 1.553075 5.275679 0.465270
C -2.094969 2.397397 0.491734
O 0.017311 1.823479 -0.410101
C -1.076643 5.046464 1.248478
H -0.984148 4.908094 2.335995
H -0.724269 6.061086 1.031627
C -2.543203 4.872536 0.847438
H -2.654596 5.098057 -0.221004
H -3.174536 5.579882 1.396326
C -3.009434 3.437540 1.116062
H -4.027398 3.310548 0.731722
H -3.072334 3.274892 2.202795
C -2.532916 1.104421 0.217394
H -1.804195 0.434676 -0.219272
C -3.832762 0.597845 0.485378
H -4.541147 1.279791 0.944815
C 9.493827 -2.836442 1.757553
H 9.768124 -3.404286 0.862534
H 10.032468 -1.874890 1.730145
H 9.840841 -3.402424 2.623785
C 7.491089 -2.615882 3.188660
H 7.452667 -1.591223 3.594211
H 6.475365 -3.024288 3.203444
H 8.101527 -3.228692 3.854262
C -4.276547 -0.676061 0.232183
C -5.769604 -2.427843 0.131297
C -4.593112 -2.925877 -0.448838
C -6.923221 -3.207240 0.223260

C -4.548337 -4.223006 -0.930983
C -6.864350 -4.517507 -0.269496
H -7.840810 -2.820889 0.653983
C -5.695641 -5.028647 -0.838527
H -3.638459 -4.614643 -1.378681
H -7.750834 -5.142537 -0.207578
H -5.674276 -6.046989 -1.214341
N -5.559011 -1.116915 0.558730
C -6.599030 -0.285705 1.139354
H -7.290164 -0.939612 1.679745
H -6.141420 0.367529 1.889543
C -7.356002 0.541322 0.093420
H -6.634896 1.149740 -0.465218
H -7.817743 -0.143674 -0.628456
C -8.419568 1.434925 0.732837
H -8.957670 2.011770 -0.026476
H -9.155687 0.839815 1.286505
H -7.967165 2.144750 1.435633
C -3.523088 -1.844313 -0.426825
C -3.082264 -1.506300 -1.867491
H -3.938074 -1.174344 -2.463438
H -2.326308 -0.717111 -1.882833
H -2.657371 -2.396506 -2.343642
C -2.317605 -2.303166 0.422931
H -1.549293 -1.527763 0.481773
H -2.635631 -2.549810 1.440692
H -1.867966 -3.197623 -0.022003
N 5.452039 0.200277 -2.289541
H 5.828627 -0.020843 -3.205153
N 4.051426 0.193377 -2.339168
H 3.644529 -0.727173 -2.174531

Int2-HDSF

G(H_2O) = -2231.075961 Hartree

C 5.346955 -0.907435 1.157809
C 4.616473 -0.495248 0.043211
C 5.255912 -0.361158 -1.197092
C 6.627051 -0.654420 -1.294015
C 7.331936 -1.066782 -0.169475
H 3.561900 -0.274138 0.142860
H 7.134364 -0.554422 -2.247644
C 2.498920 1.146577 -1.776781
C 1.151365 0.966233 -1.423948
C 3.109621 2.393221 -1.576472
C 0.438937 2.028648 -0.888246
H 0.656247 0.010122 -1.565367
C 2.375229 3.442559 -1.032448
H 4.151242 2.523895 -1.843427
C 1.024784 3.296019 -0.672925
H 2.854126 4.405219 -0.874047
C 0.203960 4.327481 -0.099969
C -1.666233 2.791186 -0.009903
C -1.108106 4.068347 0.229870
H 0.631882 5.309868 0.077738
C -2.993457 2.408964 0.277287
O -0.870659 1.793706 -0.570685
C -1.996881 5.108944 0.870714
H -1.903565 5.043917 1.964957
H -1.653031 6.109849 0.586911
C -3.461777 4.896123 0.481837
H -3.575092 5.051938 -0.598737
H -4.099403 5.631304 0.984895
C -3.914668 3.476997 0.841314
H -4.933043 3.317805 0.470782
H -3.970331 3.381483 1.936360
C -3.425150 1.102443 0.065143
H -2.692791 0.415716 -0.337579
C -4.724089 0.604454 0.351724
H -5.433279 1.301458 0.786464
C -5.167244 -0.677851 0.142822
C -6.661391 -2.430685 0.100776
C -5.487889 -2.946766 -0.469323
C -7.815406 -3.205337 0.221175
C -5.446625 -4.257763 -0.912910
C -7.760198 -4.529652 -0.232877
H -8.730507 -2.805008 0.644315
C -6.594516 -5.058678 -0.791694
H -4.539196 -4.663997 -1.352383
H -8.647054 -5.151518 -0.148733
H -6.575974 -6.087664 -1.137456
N -6.447805 -1.107525 0.487914
C -7.482745 -0.259623 1.053593
H -8.169989 -0.897682 1.617328
H -7.017835 0.413603 1.781387
C -8.247717 0.538923 -0.008564
H -7.530750 1.128612 -0.592078
H -8.718861 -0.165334 -0.705393

C	-9.302065	1.454117	0.615388
H	-9.846311	2.010192	-0.154953
H	-10.034118	0.877887	1.193818
H	-8.840212	2.182749	1.292308
C	-4.416660	-1.866123	-0.482901
C	-3.979712	-1.570554	-1.934026
H	-4.837690	-1.260099	-2.538363
H	-3.227182	-0.779015	-1.974146
H	-3.551833	-2.473060	-2.383579
C	-3.208986	-2.301353	0.376168
H	-2.437286	-1.527656	0.406367
H	-3.523103	-2.514040	1.402738
H	-2.764866	-3.211323	-0.041970
N	4.576669	0.099752	-2.322536
H	4.998109	-0.114564	-3.217418
N	3.186506	0.069098	-2.364541
H	2.792699	-0.843236	-2.140343
C	6.705773	-1.197367	1.072734
H	7.262401	-1.516047	1.944485
C	8.780212	-1.446568	-0.303853
C	4.652499	-0.967883	2.489622
F	4.607098	0.250138	3.082904
F	3.372171	-1.388615	2.373595
F	5.274105	-1.800108	3.353095
F	8.928980	-2.775452	-0.525551
F	9.482531	-1.159942	0.815222
F	9.381709	-0.809985	-1.332938

TS-HDMA

G(H_2O) = -1690.688989 Hartree

N	4.744685	-3.575204	0.389076
N	4.832709	-3.079951	-1.537280
C	3.894463	-0.340596	2.013724
C	4.648977	-1.372878	1.489404
C	4.059008	-2.580846	1.043373
C	2.653065	-2.692062	1.186455
C	1.887154	-1.676512	1.725907
C	2.483117	-0.449017	2.124113
H	4.395087	0.564588	2.333694
H	5.724818	-1.251515	1.401380
H	2.174970	-3.606832	0.846104
H	0.816369	-1.812353	1.810082
N	1.726603	0.588199	2.604168
C	4.547566	-1.743283	-1.587296
C	3.224924	-1.231477	-1.536218
C	5.620714	-0.812764	-1.511914
C	3.009476	0.116179	-1.350902
H	2.375380	-1.904487	-1.573636
C	5.388705	0.541099	-1.375853
H	6.634320	-1.201360	-1.547739
C	4.072654	1.049895	-1.255299
H	6.220747	1.237502	-1.315149
C	3.746707	2.411253	-1.012586
C	1.404545	1.831139	-0.860144
C	2.426359	2.786713	-0.783874
H	4.541066	3.149763	-0.953573
C	0.037370	2.064890	-0.560467
O	1.706153	0.533313	-1.239630
C	2.057808	4.199790	-0.394962
H	2.196877	4.323953	0.689326
H	2.747618	4.902256	-0.876677
C	0.605721	4.522365	-0.755231
H	0.496718	4.532400	-1.847334
H	0.339566	5.521105	-0.392720
C	-0.345635	3.476765	-0.162107
H	-1.370306	3.689272	-0.484938
H	-0.344226	3.566791	0.935121
C	-0.872135	1.022265	-0.585962
H	-0.480684	0.058565	-0.881036
C	-2.241313	1.098456	-0.210920
H	-2.612853	2.063997	0.116422
C	0.290071	0.432096	2.775986
H	-0.224347	0.298159	1.817594
H	0.061159	-0.424450	3.421777
H	-0.108559	1.328762	3.251265
C	2.338601	1.886509	2.840048
H	3.024510	1.867871	3.698297
H	2.897336	2.222784	1.959383
H	1.555706	2.617002	3.044616
C	-3.122154	0.046998	-0.191847
C	-5.131064	-1.041042	0.096130
C	-4.262833	-2.021490	-0.404408
C	-6.468639	-1.322453	0.373142
C	-4.718241	-3.310918	-0.622104
C	-6.917014	-2.630598	0.147497
H	-7.148328	-0.562725	0.743918
C	-6.059630	-3.618899	-0.341402

H	-4.049659	-4.076039	-1.008129
H	-7.955073	-2.873685	0.356004
H	-6.430576	-4.625272	-0.509446
N	-4.435928	0.161500	0.245846
C	-5.051474	1.393838	0.708844
H	-5.866888	1.127072	1.387815
H	-4.316069	1.941427	1.307438
C	-5.574744	2.268122	-0.436345
H	-4.749400	2.480225	-1.126386
H	-6.320754	1.696784	-1.002403
C	-6.182662	3.573849	0.077476
H	-6.555338	4.186448	-0.749890
H	-7.021789	3.379932	0.756234
H	-5.439383	4.165410	0.625352
C	-2.887417	-1.410882	-0.627200
C	-2.502709	-1.508946	-2.119398
H	-3.247175	-1.004107	-2.742692
H	-1.527143	-1.057077	-2.315783
H	-2.459175	-2.560778	-2.421476
C	-1.838998	-2.120280	0.258369
H	-0.839964	-1.701670	0.114804
H	-2.101282	-2.029942	1.317117
H	-1.799981	-3.184862	0.004186
H	5.746064	-3.389115	0.465040
H	3.972867	-3.607803	-1.693761

TS-HDSF

G(H_2O) = -2231.043842 Hartree

N	4.683650	-3.510754	-0.538581
N	4.081949	-3.025709	-2.303852
C	4.617687	-0.281457	1.296668
C	5.127205	-1.370045	0.596870
C	4.309600	-2.484301	0.303670
C	2.976370	-2.459446	0.763986
C	2.472449	-1.350148	1.432592
H	6.153966	-1.357223	0.247414
H	2.334610	-3.300839	0.527925
C	3.703435	-1.710501	-2.289090
C	2.405172	-1.273972	-1.911228
C	4.711339	-0.724803	-2.490189
C	2.167686	0.063905	-1.682559
H	1.611080	-1.990701	-1.732013
C	4.452482	0.610762	-2.284267
H	5.700778	-1.062183	-2.783114
C	3.181127	1.046990	-1.831368
H	5.235815	1.350987	-2.414431
C	2.870344	2.380461	-1.483208
C	0.622683	1.719554	-0.878834
C	1.611843	2.707862	-0.990390
H	3.633206	3.147342	-1.575689
C	-0.708733	1.946402	-0.438687
O	0.915538	0.417649	-1.244376
C	1.275996	4.115656	-0.561635
H	1.514507	4.238276	0.505219
H	1.910027	4.823916	-1.106197
C	-0.206183	4.415621	-0.786151
H	-0.418756	4.398201	-1.862740
H	-0.451850	5.418933	-0.422115
C	-1.079520	3.375448	-0.078397
H	-2.128184	3.559334	-0.331349
H	-0.997685	3.514337	1.010194
C	-1.624203	0.909229	-0.346039
H	-1.268470	-0.079229	-0.606130
C	-2.975130	1.062687	0.066844
H	-3.269575	2.050051	0.403584
C	-3.960584	0.105384	0.063149
C	-6.093211	-0.735769	0.268571
C	-5.345171	-1.790417	-0.270511
C	-7.462254	-0.852452	0.503728
C	-5.957405	-2.995846	-0.571184
C	-8.070481	-2.075563	0.192325
H	-8.046628	-0.031808	0.905530
C	-7.334831	-3.138385	-0.337195
H	-5.383424	-3.820491	-0.985647
H	-9.136583	-2.191966	0.364822
H	-7.828941	-4.076049	-0.572231
N	-5.251872	0.358369	0.499027
C	-5.734625	1.641431	0.985642
H	-6.575841	1.447594	1.657693
H	-4.948815	2.096646	1.596012
C	-6.160222	2.584170	-0.145927
H	-5.311997	2.727601	-0.825772
H	-6.953027	2.098514	-0.727868
C	-6.643818	3.932394	0.389342
H	-6.947526	4.593641	-0.428679
H	-7.504312	3.807136	1.057411
H	-5.852336	4.438662	0.954609

C	-3.894074	-1.357154	-0.406587
C	-3.427348	-1.484041	-1.870676
H	-4.042232	-0.861444	-2.527765
H	-2.383391	-1.184810	-1.990666
H	-3.522041	-2.525023	-2.196942
C	-3.007601	-2.200141	0.535572
H	-1.969698	-1.867611	0.514240
H	-3.370039	-2.131806	1.566259
H	-3.040730	-3.251945	0.231115
H	5.666457	-3.397507	-0.789919
H	3.257055	-3.619841	-2.211206
C	3.280841	-0.245495	1.700574
H	2.878611	0.626160	2.200599
C	1.019482	-1.331651	1.803864
C	5.479441	0.930931	1.505114
F	6.785058	0.608476	1.642882
F	5.402597	1.793660	0.460140
F	5.116375	1.627868	2.605802
F	0.268868	-1.922684	0.836498
F	0.544856	-0.079683	1.968863
F	0.764118	-2.005989	2.950300

References:

1. He, X., Li, L., Fang, Y., Shi, W., Lia, X., Ma, H. In vivo imaging of leucine aminopeptidase activity in drug-induced liver injury and liver cancer via a near-infrared fluorescent probe. *Chem. Sci.* **8**, 3479-3483 (2017).
2. Tian, X., Li, Z., Sun, Y., Wang, P., Ma, H. Near-Infrared Fluorescent Probes for Hypoxia Detection via Joint Regulated Enzymes: Design, Synthesis, and Application in Living Cells and Mice. *Anal. Chem.* **90**, 13759-13766 (2018).
3. Frisch, M. J.; Trucks, G. W.; Schlegel, H. B.; Scuseria, G. E.; Robb, M. A.; Cheeseman, J. R.; Scalmani, G.; Barone, V.; Mennucci, B.; Petersson, G. A.; Nakatsuji, H.; Caricato, M.; Li, X.; Hratchian, H. P.; Izmaylov, A. F.; Bloino, J.; Zheng, G.; Sonnenberg, J. L.; Hada, M.; Ehara, M.; Toyota, K.; Fukuda, R.; Hasegawa, J.; Ishida, M.; Nakajima, T.; Honda, Y.; Kitao, O.; Nakai, H.; Vreven, T.; Montgomery, J. A., Jr.; Peralta, J. E.; Ogliaro, F.; Bearpark, M.; Heyd, J. J.; Brothers, E.; Kudin, K. N.; Staroverov, V. N.; Keith, T.; Kobayashi, R.; Normand, J.; Raghavachari, K.; Rendell, A.; Burant, J. C.; Iyengar, S. S.; Tomasi, J.; Cossi, M.; Rega, N.; Millam, J. M.; Klene, M.; Knox, J. E.; Cross, J. B.; Bakken, V.; Adamo, C.; Jaramillo, J.; Gomperts, R.; Stratmann, R. E.; Yazyev, O.; Austin, A. J.; Cammi, R.; Pomelli, C.; Ochterski, J. W.; Martin, R. L.; Morokuma, K.; Zakrzewski, V. G.; Voth, G. A.; Salvador, P.; Dannenberg, J. J.; Dapprich, S.; Daniels, A. D.; Farkas, O.; Foresman, J. B.; Ortiz, J. V.; Cioslowski, J.; Fox, D. J. Gaussian 09, revision D.01; Gaussian Inc., Wallingford, CT, 2013.
4. Becke, A. D. Density Functional Thermochemistry. III. The Role of Exact Exchange. *J. Chem. Phys.* **98**, 5648 (1993).
5. Lee, C.; Yang, W.; Parr, R. G. Development of the Colle-Salvetti Correlation-Energy Formula into a Functional of the Electron Density. *Phys. Rev. B.* **37**, 785 (1998).
6. Grimme, S., Antony, J., Ehrlich, S. and Krieg, J. A Consistent and Accurate ab Initio Parametrization of Density Functional Dispersion Correction (DFT-D) for the 94 Elements H-Pu. *J. Chem. Phys.* **132**, 154104 (2010).
7. Barone, V., Cossi, M. Quantum calculation of molecular energies and energy gradients in solution by a conductor solvent model. *J. Phys. Chem. A* **102**, 1995-2001 (1998).
8. Cossi, M., Rega, N., Scalmani, G., Barone, V. Energies, structures, and electronic properties of molecules in solution with the C-PCM solvation model. *J. Comput. Chem.* **24**, 669-681 (2003).
9. Takano, Y., Houk, K. N. Benchmarking the Conductor-like Polarizable Continuum Model (CPCM) for Aqueous Solvation Free Energies of Neutral and Ionic Organic Molecules. *J. Chem. Theory Comput.* **1**, 70-77 (2005).
