# Supplementary material for: Rational construction of a reversible arylazo-based NIR probe for cycling hypoxia imaging in vivo
Source: Nat Commun. 2021 May 13;12:2772. doi: 10.1038/s41467-021-22855-0 (PMC8119430; doi:10.1038/s41467-021-22855-0)
Supplement: Supplementary file 3 — Description of Additional Supplementary Files [file 41467_2021_22855_MOESM3_ESM.pdf]

Description of Additional Supplementary Files

File Name: Supplementary Movie 1
Description: Doppler Ultrasound imaging of the blood flow in the right hind limb before treated with a tourniquet.

File Name: Supplementary Movie 2
Description: Doppler Ultrasound imaging of the blood flow in the right hind limb when treated with a tourniquet.

File Name: Supplementary Movie 3
Description: Doppler Ultrasound imaging of the blood flow in the right hind limb 5 min post removal of the tourniquet.
