# Supplementary material for: Rational construction of a reversible arylazo-based NIR probe for cycling hypoxia imaging in vivo
Source: Nat Commun. 2021 May 13;12:2772. doi: 10.1038/s41467-021-22855-0 (PMC8119430; doi:10.1038/s41467-021-22855-0)
Supplement: Supplementary file 7 — Reporting Summary [file 41467_2021_22855_MOESM7_ESM.pdf]

nature research

Corresponding author(s): Yuncong Chen, Zijian Guo, Qingshun Zhao, Weijiang He

Last updated by author(s): Mar 23, 2021

Reporting Summary

Nature Research wishes to improve the reproducibility of the work that we publish. This form provides structure for consistency and transparency in reporting. For further information on Nature Research policies, see our Editorial Policies and the Editorial Policy Checklist.

Statistics

For all statistical analyses, confirm that the following items are present in the figure legend, table legend, main text, or Methods section.

n/a	Confirmed	
☐	☒	The exact sample size (*n*) for each experimental group/condition, given as a discrete number and unit of measurement
☐	☒	A statement on whether measurements were taken from distinct samples or whether the same sample was measured repeatedly
☐	☒	The statistical test(s) used AND whether they are one- or two-sided<br>*Only common tests should be described solely by name; describe more complex techniques in the Methods section.*
☐	☒	A description of all covariates tested
☐	☒	A description of any assumptions or corrections, such as tests of normality and adjustment for multiple comparisons
☐	☒	A full description of the statistical parameters including central tendency (e.g. means) or other basic estimates (e.g. regression coefficient) AND variation (e.g. standard deviation) or associated estimates of uncertainty (e.g. confidence intervals)
☐	☒	For null hypothesis testing, the test statistic (e.g. *F*, *t*, *r*) with confidence intervals, effect sizes, degrees of freedom and *P* value noted<br>*Give P values as exact values whenever suitable.*
☒	☐	For Bayesian analysis, information on the choice of priors and Markov chain Monte Carlo settings
☐	☒	For hierarchical and complex designs, identification of the appropriate level for tests and full reporting of outcomes
☐	☒	Estimates of effect sizes (e.g. Cohen's *d*, Pearson's *r*), indicating how they were calculated

Our web collection on statistics for biologists contains articles on many of the points above.

Software and code

Policy information about availability of computer code

Data collection	Bruker DRX-400, Agilent 6540Q-TOF LC/MS mass spectrometer, Thermo Fisher ESI Mass Spectrometer, FluoroMax-4 Spectrofluorometer, Perkin Elmer Lambda 35 spectrophotometer, confocal microscope Zeiss LSM710, PerkinElmer IVIS Lumina K Series III in vivo imaging system, VisualSonics Vevo2100
Data analysis	Average area intensity in cells were given by the Zen software on confocal microscope Zeiss LSM710. Average area intensity in mice were given by the Living Image software on PerkinElmer IVIS Lumina K Series III in vivo imaging system. All data were analyzed and calculated with Microsoft Excel 2016 software (Microsoft, Redmond, WA), and the statistical differences were analyzed by two-tailed student's test. All statistical data are presented as means ± SD. All statistical graph and fluorescent spectra were performed using Origin 8.5 (OriginLab Corporation, MA, USA). Quantum chemistry calculation was performed with Gaussian 0.9, revision D.01 (Gaussian Inc., Wallingford, CT, 2013).

For manuscripts utilizing custom algorithms or software that are central to the research but not yet described in published literature, software must be made available to editors and reviewers. We strongly encourage code deposition in a community repository (e.g. GitHub). See the Nature Research guidelines for submitting code & software for further information.

Data

Policy information about availability of data

All manuscripts must include a data availability statement. This statement should provide the following information, where applicable:

- Accession codes, unique identifiers, or web links for publicly available datasets
- A list of figures that have associated raw data
- A description of any restrictions on data availability

The authors declare that all data supporting conclusions of this work are available either in the paper or in the supporting information files or from the authors upon requested. Source data are provided as a source data file with this paper, underlying Fig. 1c-f, Fig. 4e-f, Fig. 6c-d, Fig. 7n, Supplementary Fig. 8a-c, Supplementary

Fig. 9, Supplementary Fig. 10, Supplementary Fig. 12, Supplementary Fig. 13, Supplementary Fig. 14, Supplementary Fig. 15, Supplementary Fig. 16, Supplementary Fig. 18, Supplementary Fig. 19b, Supplementary Fig. 22, and Supplementary Fig. 24.

Field-specific reporting

Please select the one below that is the best fit for your research. If you are not sure, read the appropriate sections before making your selection.

[x] Life sciences [ ] Behavioural & social sciences [ ] Ecological, evolutionary & environmental sciences

For a reference copy of the document with all sections, see nature.com/documents/nr-reporting-summary-flat.pdf

Life sciences study design

All studies must disclose on these points even when the disclosure is negative.

Sample size	Sample size (n) is supplied in figure legends in main paper or the supporting information file. All sample size was determined by number of the biological replicate necessary to ensure statistical significance, so as to support meaning conclusions, and based on the sample number from preliminary studies and similar studies (Zhou, H. et al. Nat. Commun. 2020, 11, 6183; Fang, H. et al. Nat. Commun. 2021, 12, 109.).
Data exclusions	No data were excluded.
Replication	All data presented were repeated for at least three times with similar results. All replicating attempts were successful.
Randomization	All samples were randomly divided into experimental groups.
Blinding	Data collection were performed automatically with machine setups independently to the observer. For imaging analysis in cells and mice, independent third individuals were invited to assess biological effects.

Reporting for specific materials, systems and methods

We require information from authors about some types of materials, experimental systems and methods used in many studies. Here, indicate whether each material, system or method listed is relevant to your study. If you are not sure if a list item applies to your research, read the appropriate section before selecting a response.

Materials & experimental systems

n/a	Involved in the study	
[x]	[ ]	Antibodies
[ ]	[x]	Eukaryotic cell lines
[x]	[ ]	Palaeontology and archaeology
[ ]	[x]	Animals and other organisms
[x]	[ ]	Human research participants
[x]	[ ]	Clinical data
[x]	[ ]	Dual use research of concern

Methods

n/a	Involved in the study	
[x]	[ ]	ChIP-seq
[x]	[ ]	Flow cytometry
[x]	[ ]	MRI-based neuroimaging

Eukaryotic cell lines

Policy information about cell lines

Cell line source(s)	The human breast carcinoma cell line MCF-7 were purchased from National Collection of Authenticated Cell Cultures.
Authentication	Cell lines were authenticated by short tandem repeat (STR) testing.
Mycoplasma contamination	Cells lines were tested negative for mycoplasma contamination.
Commonly misidentified lines (See ICLAC register)	No commonly misidentified cell lines were used.

Animals and other organisms

Policy information about studies involving animals; ARRIVE guidelines recommended for reporting animal research

Laboratory animals	Eight-week-old female ICR mice, NCG MCF-7 tumor-bearing female mice, and male adult nude mice were all obtained from the Model Animal Research Center of Nanjing University (NJU). Mice were housed in polycarbonate cages with 22-25 celsius degree, 35-45% humidity, 12 h light-darkness cycles, and standard food and water supply. Cages were cleaned at regular interval. All Transgenic zebrafish embryos used in co-localization tests were obtained from China Zebrafish Resource Center. Other zebrafish embryos used in cycling hypoxia imaging tests were wild type Tubingen strain (TU), gifts from Zhao Qingshun Group of the Model

	Animal Research Center (NJU). All zebrafish were kept in in E3 medium at 28 celsius degree.
Wild animals	The work did not involve wild animals.
Field-collected samples	The work did not involve field-collected samples.
Ethics oversight	All animal tests were conducted according to the Guidelines for the Care and Use of Laboratory Animals of the Chinese Animal Welfare Committee and approved by the Institutional Animal Care and Use Committee of Nanjing University.

Note that full information on the approval of the study protocol must also be provided in the manuscript.
